# Antimicrobial peptides: mechanism of action, activity and clinical potential

**DOI:** 10.1186/s40779-021-00343-2

**Published:** 2021-09-09

**Authors:** Qi-Yu Zhang, Zhi-Bin Yan, Yue-Ming Meng, Xiang-Yu Hong, Gang Shao, Jun-Jie Ma, Xu-Rui Cheng, Jun Liu, Jian Kang, Cai-Yun Fu

**Affiliations:** 1 Zhejiang Provincial Key Laboratory of Silkworm Bioreactor and Biomedicine, College of Life Sciences and Medicine, Zhejiang Sci-Tech University, No. 928, Street 2, Xiasha Higher Education Zone, Hangzhou, 310018 Zhejiang China; 2 Department of Oncology, The 903rd Hospital of PLA, Hangzhou, 310013 Zhejiang China; 3 Department of Pharmaceutical Chemistry and the Cardiovascular Research Institute, University of California San Francisco, 555 Mission Bay Blvd. South, San Francisco, CA 94158 USA; 4 Oncogenic Signaling and Growth Control Program, Peter MacCallum Cancer Centre, 305 Grattan Street, Melbourne, VIC 3000 Australia; 5 Sir Peter MacCallum Department of Oncology, University of Melbourne, Parkville, VIC 3010 Australia

**Keywords:** Antimicrobial peptides, Antimicrobial resistance, Mechanism of action, Biological activity, Clinical application

## Abstract

The management of bacterial infections is becoming a major clinical challenge due to the rapid evolution of antibiotic resistant bacteria. As an excellent candidate to overcome antibiotic resistance, antimicrobial peptides (AMPs) that are produced from the synthetic and natural sources demonstrate a broad-spectrum antimicrobial activity with the high specificity and low toxicity. These peptides possess distinctive structures and functions by employing sophisticated mechanisms of action. This comprehensive review provides a broad overview of AMPs from the origin, structural characteristics, mechanisms of action, biological activities to clinical applications. We finally discuss the strategies to optimize and develop AMP-based treatment as the potential antimicrobial and anticancer therapeutics.

**Supplementary Information:**

The online version contains supplementary material available at 10.1186/s40779-021-00343-2.

## Background

Antimicrobial peptides (AMPs) are the small molecular peptides that play a crucial role in the innate immunity of the host [1] against a broad range of microorganisms, including bacteria, fungi, parasites and viruses [2–4]. To date, the AMP database [Data Repository of Antimicrobial Peptides (DRAMP), http://dramp.cpu-bioinfor.org/] has reported 3791 AMPs from six kingdoms, including 431 from bacteria, 4 from archaea, 7 from protozoal, 6 from fungal, 824 from plants and 2519 from animals [5]. Besides antibacterial activities, AMPs have been found to possess a variety of biological functions, such as immune regulation, angiogenesis, wound healing and antitumor activity [6–9]. The treatment of pathogenic bacteria has been long-time mainly relied on antibiotics. However, the emergence of drug resistance due to the single target of antibiotics, long-term and extensive utilization, is becoming a major challenge for clinical infection management [10, 11]. In contrast, AMPs show the advantages by acting on multiple targets on the plasma membrane and intracellular targets of pathogenic bacteria, and have potent activity on drug-resistant bacteria [4, 12, 13]. Thus, AMPs provide a new alternative to antibiotics. Furthermore, the long-term chemotherapy in cancer patients not only leads to resistance to conventional cancer treatments, but also results in the susceptibility to pathogenic infection. AMPs have antibacterial and anticancer properties, and thus is a new treatment option for cancer patients. At present, the clinical application of AMPs is mainly on the treatment of pathogenic bacteria infection, wound healing and inflammation [14, 15]. While a few AMPs have entered the clinical stage of cancer treatment, the inevitable defects in the natural AMPs are the obstacles to development of AMPs with therapeutic efficacy. Therefore, to overcome these shortcomings, it is essential to further explore the structural characteristics and mechanism of action of AMPs to improve their stability, activity, targeting, and reduction of cytotoxicity. This review extensively overviews the origin, structural characteristics, mechanisms of action and biological activity of AMPs with the aim to provide the comprehensive current knowledge and understanding of AMPs and more importantly, the new prospects for clinical development and applications of AMPs.

## Natural distribution of AMPs

As an ancient host defense mechanism against pathogen invasion, AMPs are well preserved in eukaryotes. This is because that: (1) as a component of the innate immune system, production of AMPs by the host cells requires less time and energy than antibody synthesis by the acquired immunity; (2) these small molecule peptides can reach the target faster than immunoglobulins; and (3) some eukaryotes lack of lymphocyte-based immune system, such as insects mainly rely on the synthesis of a series of antibacterial compounds to remove invading microorganisms [16]. Since the first AMP was discovered in the American silkworm chrysalis, a large number of AMPs have been widely found in various organisms, including microorganisms [17], plants [18], invertebrates [19], fish [20], amphibians [21], reptiles [22], birds [23] and mammals [24].

The first AMP isolated in bacteria is nisin, which produced by the host strain has cytotoxicity to other types of bacteria in order to compete for nutrients in the environment [25]. In recent decades, nisin has been widely used as a natural preservative in many foods due to its antiseptic activity [26–28]. AN5-1 was originally isolated from the fermentation broth of *Paenibacillus alvei* strain [29]. It destroys the bacterial membrane and inhibits cellular functions by integrating and disrupting the bacterial genomic DNA [30]. Besides, recent studies reported that intestinal microbiota served as a source of AMPs [31, 32]. AMPs have also been found in fungi [17]. In addition, Copsin originated from *Coprinopsis cinerea* (*Mushroom*), has bactericidal effects on a variety of Gram-positive bacteria by interfering with the biosynthesis of the cell wall of pathogens, such as *Enterococcus faecalis* (*E*. *faecalis*) and *Listeria monocytogenes* (*L. monocytogenes*) [33].

AMPs also protect plants from the invasion of pathogenic microorganisms in the air and soil. There are multiple families of plants-derived AMPs, including thionins, defensins and cyclotides [34]. Thionins are widely found in seeds, stems, roots and leaves of plants [35] and have cytotoxic effects on Gram-positive bacteria [36], Gram-negative bacteria [36], yeasts [37] and other fungi [38]. Plants-derived AMPs are usually rich in cysteine residues to form multiple disulfide bonds that are important for structural stabilization [39].

Due to lack of lymphocyte-based immune system, invertebrates mainly rely on the innate immune system as the first line of host defense to resist the invasion of pathogenic bacteria [40]. Invertebrate AMPs are widely distributed in hemolymph, mucosa of skin and other tissues. For example, cecropins derived from hemolymph of *Hyalophora cecropia*, have a strong antibacterial effect on Gram-positive and Gram-negative bacteria [41]. The induced expression of drosocin in the intestinal tract of drosophila can prevent the infection of pathogen *Pseudomonas entomophila* [42] and thus maintain intestinal homeostasis. The Toll and Imd pathways are the important pathways in regulation of AMP production in drosophila [43], and the similar regulatory pathways have also been found in mammals [44].

Together with inorganic substances (hydrogen peroxide and nitric oxide), antibacterial proteins (such as lysozyme, azurocidin, cathepsin G, phospholipase A_2_ and lactoferrin), AMPs constitute the innate immune system of mammals [45]. To date, more than 1770 species of AMPs have been found in vertebrates. Most mammals mainly have the two classes of AMPs termed cathelicidins and defensins [46, 47], and fish also contains hepcidins and piscidins [48].

Cathelicidins are a class of AMPs which have a highly conserved cathelin domain and the distinct peptide lengths, amino acid sequences and protein structures [49]. They are stored in a nonfunctional form in neutrophils and macrophage secretory granules and become activated after being processed and released upon leukocyte activation [46]. Cathelicidins (CATH BRALE and codCath1) derived from fish show potential antibacterial activity to a broad spectrum of Gram-positive and Gram-negative bacteria [50]. Skin is an important source of AMPs for amphibian [51]. Cathelicidin-PV, an AMP identified in the skin of the frog *Paa yunnanensis*, has strong antibacterial activity against Gram-positive and Gram-negative bacteria, fungi, as well as clinically isolated drug-resistant and standard strains but has low hemolytic activity [52]. Cathelicidin-related peptide (crotalicidin) has been identified in the rattlesnake of South America. Both crotalicidin and its fragments (15–34) have potential antibacterial, anti-tumor and anti-fungal properties [53]. These peptides killed 90% of *Escherichia coli* (*E. coli*) and *Pseudomonas aeruginosa* (*P. aeruginosa*) cells within 90–120 min and 5–30 min, respectively. In addition, cathelicidins are also found in birds [54], cattle [55], horses [56], pigs [57], goats [58–60], sheep [61], chickens [62, 63], dogs [64] and rabbits [65]. Notably, LL-37 is the only cathelicidin that is found in humans [66].

Another group of AMPs is defensins. They are divided into three subtypes based on the arrangement of disulfide bonds including α-, β- and θ-defensins [67, 68]. α-defensins and θ-defensins evolved from an ancient β-defensins [69]. In humans, there are only α-defensins and β-defensins but no θ-defensins due to an early termination codon in the mRNA. Reptiles and birds only produce β-defensins while θ-defensins are found in the leukocytes and bone marrow of some non-human primates [46, 47, 69]. Similar to cathelicidins, α-defensins are cleaved by elastase, metalloproteinase or other proteolytic enzymes and ultimately formed a C-terminal peptide with potential antimicrobial activity [70]. The first β-defensin is found in the epithelial cells of cattle [71]. Turtle β-defensin 1 (TBD-1) is the first β-defensin isolated from leukocytes in reptiles with a high homology to β-defensins from birds and mammals [72]. Wang et al*.* [73] showed that 11 bacteria (including Gram-positive and Gram-negative bacteria) were almost completely killed by AvBD9, a kind of β-defensins derived from quail, at the concentration of 25 μg/ml.

As the key component in the innate immunity, AMPs are generated in the sites where the body is most vulnerable to pathogen invasion. In mammals, the AMPs-rich mucus resists the colonization of parasites, bacteria and fungi [74, 75]. AMPs are also found in phagocytic granulocytes and mast cells [76]. The α-defensins in mammals mainly exist in neutrophils, macrophages and intestinal Paneth cells, while β-defensins exist more extensively including leukocytes and epithelial cells in the skin, the respiratory, digestive and genitourinary tracts, as well as the blood and urine [77]. The human β-defensin 3 (HBD3) also exists in the heart and skeletal muscle [77]. While the eyes are always exposed to the outside and at the risk of pathogenic bacterial infection at all times. The AMPs in eyes play a key role in infection prevention [2]. Cathelicidins are originally isolated from bone marrow cells [78]. Similar to defensins, most cathelicidins are stored in the granules of neutrophils or macrophages and can be secreted by epithelial cells and immune cells [46, 79] and widely distributed in mucosal secretions, blood, urine, sweat and tears [80–83]. Characterization of the structure and physiochemical features of AMPs can help us to identify the novel AMPs. The new technologies, such as the new genome mining approaches using machine learning and sequence-based encodings [84] will accelerate this discovery process.

## Structure and characteristics of AMPs

AMPs are divided into several subgroups on the basis of amino acid sequences, the net charge of the peptide, protein structure and sources (Additional file 1: Table S1). Most AMPs have a net charge of + 2 to + 9 and contain 10–100 amino acids [85]. The Database of Antimicrobial Activity and Structure of Peptides (DBAASP, https://dbaasp.org/) is an open-access, comprehensive database containing information related to amino acid sequences, chemical modifications, 3D structures, bioactivities and toxicities of peptides that possess antimicrobial properties. The latest version 3.0 (DBAASP v3) contains > 15,700 entries (8000 more than the previous version) [86].

The first subgroup is the anionic AMPs which have a net charge range of − 1 to − 8 and contain 5 to 70 amino acid residues [87]. The majority of anionic AMPs are the peptide fragments after proteolysis but some anionic AMPs are the small molecules encoded by genes. Their structure features include α-helical peptides from some amphibians and cyclic cystine knots [87]. They seem to utilize metal ions and the negatively charged components of the microbial membrane to form salt bridges, thus interacting with microbes [88], which are similar to the charge-neutralization characteristics of larger proenzyme [89]. For example, ovine pulmonary surfactant associated anion peptide (SAAP), the first discovered anionic AMP with 5–7 aspartate residues, had antimicrobial activity to the ovine pathogen *Mannheimia haemolytica* in the presence of Zn ions [90]. When 0.14 mol/L NaCl and EDTA were added into the surfactant solution, its bactericidal activity was largely inhibited, while restored when ZnCl_2_ was replenished. In addition, the amidated C-terminal fragment of the α-helical anionic AMP maximin H5 forms an intra-peptide hydrogen bond with the N-terminal region of the peptide, important for stabilizing the tilted α-helix structure [87].

The second subgroup is the cationic α-helical AMPs. These small peptides with less than 40 amino acids in length, carry a net charge of + 2 to + 9 and mostly have the C-terminus amidated [91]. The structure of these peptides is disordered in aqueous solutions, but in the presence of trifluoroethanol, sodium dodecyl sulfate (SDS) micelles, phospholipid vesicles, and liposomes or liposomes A, the molecules are all or partly transformed into α-helical structure [92]. In addition, these AMPs usually contain over 50% hydrophobic amino acids, which enable the formation of amphiphilic structure when interacting with target cells [93]. Most cathelicidins are amphiphilic α-helical AMPs [6], in which cecropins, magainins and LL-37 have been well studied. LL-37 is the only human cathelicidin of an active fragment released from hCAP18 by serine protease 3 in neutrophils with a net charge of + 6 at a neutral pH [94, 95]. The circular dichroism of LL-37 shows a disordered structure in water and is transformed into an α-helical structure in the presence of HCO_3_^−^, SO_4_^2−^, or CF_3_CO_2_^−^ at the concentration of 15 mmol/L [96]. The efficiency of structural transformation is directly proportional to the concentrations of the peptide. Moreover, the degree of α-helix is correlated with the antibacterial activity of LL-37 against Gram-positive and Gram-negative bacteria.

The third subgroup is the cationic β-sheet AMPs. The peptides typically contain 2–8 cysteine residues forming 1–4 pairs of intramolecular disulfide bonds [97]. The disulfide bonds are essential for structure stabilization and biological functions of these peptides. For example, they become inactivated when cysteines are replaced by acidic amino acids, while remain active when mutating to hydrophobic amino acids (excluding alanine and leucine) [98]. However, the structure and disulfide bonds of human neutrophilic peptide 1 (HNP1), HBD3 and mouse defensins are not required for antimicrobial activity or cytotoxicity [70, 99]. The β-sheet AMPs consist primarily of defensins [97]. As mentioned above, the mammalian defensins are classified as α-defensins and β-defensins according to the characteristic intervals between the six cysteine and disulfide bond modes [100]. Despite difference in covalent structures, the mammalian defensins display very similar tertiary structures [101]. In the case of α-defensins, near the amino terminus they form a three-stranded chain by hydrogen bonding with the β-hairpin, and a cyclic structure by pairing cysteine with disulfide bonds [101]. The bactericidal activity of amphipathic α-defensins depends on the positive charge and hydrophobic amino acids that cause bacterial membrane destruction by interacting with phosphatidyl chains [102]. Moreover, the interaction between cationic α-defensin residues and negatively charged substances on the bacterial surface may precede the interaction between hydrophobic residues and the membranes and thus primarily mediate membrane destruction and bacterial killing. In the case of β-defensins, some defensins contain both α-helix and β-sheet. For instance, the insect defensin A, has an α-helix of 11 amino acids in the middle (residues 14–24), and its N-terminal β-hairpin is parallel to the α-helix with a cyclic structure formed by the first 13 amino acid residues [103]. The antibacterial and antiparasitic activities are predominantly mediated by the N-terminal domain of the chicken Gga-AvBD11 and enhanced by its C-terminal domain while the antiviral activity requires the full-length protein [104]. The θ-defensins are the end-to-end cyclized tetracyclic peptides that have three disulfide bridges to connect their antiparallel β-sheets [105]. The cyclic structure of the θ-defensins allows them to remain active at high concentration of salt and is essential for their antimicrobial properties supported by decrease of the microbicidal activities caused due to loss of the cyclic structure [106]. Recent studies further reveal that the structure and stability of defensins mainly depend on the number and position of the disulfide bonds, while their antibacterial and membrane-binding properties rely on the cyclic backbone [107].

The fourth subgroup is the extended cationic AMPs containing the specific amino acids including arginine, proline, tryptophan, glycine and histidine, but lacks regular secondary structures [93]. Their structures are stabilized only by hydrogen bonds and van der Waals force of interacting with the membrane lipids. Typically, PR-39 is rich in proline (49%) and arginine (24%) [108], prophenin-1 is rich in proline (53.2%) and phenylalanine (19%) [109], indolicidin is rich in tryptophan (38%) and proline (23%) [110], and histatin-8 is rich in histidine (33.3%) [111].

The fifth subgroup is the fragments from antimicrobial proteins. Some naturally occurring proteins and their fragments have a broad-spectrum bactericidal effect. Lysozyme, the first discovered antimicrobial protein, is a key component of the innate immune system against foreign pathogens [112–114]. Its extracellular fragment contains 130 amino acids and has an α-helix and β-sheet structure. A helix-loop-helix (HLH) region in the lysozyme of human and chicken has been also found in other membrane active and DNA binding proteins [115]. The HLH peptide has a strong bactericidal effect against Gram-positive and Gram-negative bacteria, and the fungus *Candida albicans* (*C. albicans*). More recently, Toda et al*.* [116] identified a sleep-inducing gene in fruit flies encoding NEMURI protein which contains an arginine-rich region, and possessed immunomodulatory functions and strong bactericidal effect comparable to that of kanamycin. Other antibacterial proteins are shown in Additional file 1: Table S1.

Notably, some AMPs contain the amino terminal copper and nickel (ATCUN) binding motif. It is composed of the sequence H_2_N-XXH found in the N-terminus, where the XX can be any amino acid other than proline [117]. Cu^2+^ and Ni^2+^ can bind to the motif with a high affinity [118]. The Cu^2+^-ATCUN complex can produce reactive oxygen species (ROS) [119, 120], which target nucleic acids, proteins and lipids [118].

## Targeting specificity of AMPs

The central question in the research of AMPs is how AMPs these peptides specifically target the invading pathogen while spare the host cells? The differences in the composition of cell membrane between the pathogens and the host cells have been considered to underpin the targeting specificity of AMPs. In general, the lipids and proteins are the main components of the cellular membrane and form the phospholipid bilayer as the basic scaffold for the cell membrane. Phosphatidylcholine (PC) and phosphatidyl ethanolamine (PE) are normally uncharged, while hydroxylated phospholipids such as phosphatidylserine (PS), cardiolipin (CL) and phosphatidylglycerol (PG) are negatively charged. Intriguingly, PS, PG and CL are found in bacterial pathogens but have little or no presence in mammalian cytoplasmic membrane [121, 122]. In contrast, PE and PC are commonly found in mammalian cell membranes [122]. In addition, sterols such as cholesterol (mammalian) and ergosterol (fungi) are present in eukaryotes but rarely in prokaryotic cell membranes [123, 124]. Moreover, the lipopolysaccharide (LPS) of Gram-negative bacteria and the lipoteichoic acid of Gram-positive bacteria carry a large number of negative charges, which increases the amount of negative charge of the membrane. Different from bacteria, the negative charges of the fungal membranes mainly resulted from the phosphomannan and other related components, such as negatively charged phosphatidylinositol (PI), PS and diphosphatidylglycerol (DPG) [125]. Consequently, the cationic AMPs selectively interact with the negatively charged membrane through electrostatic interaction which partially explains the targeting specificity of AMPs.

Compared with normal cells, cancer cells also exhibit more negatively charged PS outside of membrane [126]. Furthermore, the high expression of glycoproteins that contain repeated regions of O-glycosylation [126] and some other anionic components such as gangliosides and heparan sulfates on the membrane surface of cancer cells also contribute to the negative charge on the surface [127, 128]. In addition, the presence of a large number of microvilli on the membrane surface of cancer cells increases the area available for AMPs binding [129].

## Mechanism of action of AMPs

### Membrane model

The cationic AMPs exert antibacterial activity by interacting with negatively charged bacterial membrane to increase membrane permeability and lead to cell membrane lysis and cell content release. Upon approaching the cytoplasmic membrane through electrostatic interaction with the microbial membrane, AMPs bind to the microbial membrane and interact with the anionic components of the plasma membrane. Prior to this, AMPs have to pass through the capsular polysaccharide and other components of the cell wall, such as LPS of Gram-negative bacteria and lipoteichoic acid and peptidoglycan of Gram-positive bacteria [130–132]. In this step, there are two major factors that affect the interaction, namely the conformational change and the peptide-lipid ratio [133–136]. Studies have shown that α-helical AMPs bind to the anionic lipid membrane and transformed its disordered structure in aqueous solution into the amphiphilic α-helical structure to facilitate the interaction with the membrane [137]. Different from α-helical peptides, the β-sheet peptides do not undergo a major conformational transition when interacting with the membrane due to their stable disulfide bond bridge [137]. The peptide-lipid ratio is another major factor that affects AMP interaction with cell membrane. At a low peptide-lipid ratio, AMPs are located in parallel on the surface of the plasma membrane [138, 139]. With the increase of the peptide-lipid ratio, AMPs are vertically oriented and inserted into the hydrophobic center of the membrane. Eventually, membrane permeation leads to the leakage of intracellular ions, metabolites and biosynthesis, with the consequent cell death [140].

**Fig. 1:**
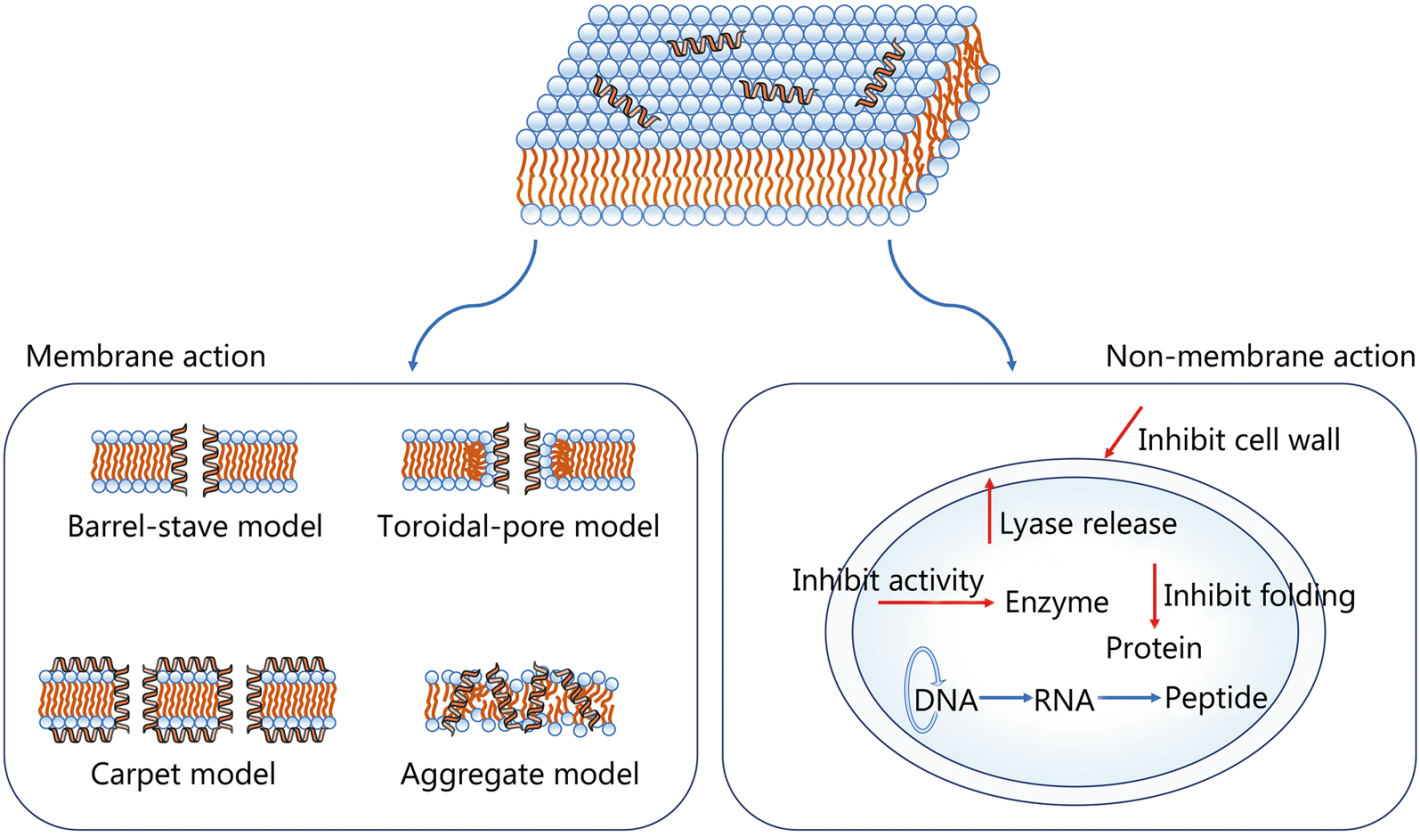
Models of antibacterial mechanisms of AMPs. The direct bactericidal mechanism of AMPs is performed through interacting with negatively charged membranes, resulting in increased membrane permeability, cell membrane lysis, or release of intracellular contents, which ultimately leads to cell death. There are four main models of membrane-pore formation, namely barrel-stave model, toroidal-pore model, carpet model and aggregate model. After AMPs penetrate into the phospholipid membrane, their hydrophobic regions combine with the internal hydrophobic regions of the phospholipid bilayer, while their hydrophilic regions are exposed to the outside. Another bactericidal mechanism is that AMPs penetrate into the cytoplasm and interact with intracellular substances, such as inhibiting DNA, RNA and protein synthesis, inhibiting protein folding, inhibiting enzyme activity and cell wall synthesis, and promoting the release of lyases to destroy cell structures. AMPs antimicrobial peptides

Some hypothetical models of membrane-cavity formation, such as barrel-stave, toroidal-pore, carpet and aggregate models, have been proposed (Fig. 1). In the barrel-stave model, with the increased amounts of peptide binding to the membrane, aggregation and conformational transformation occur, which causes local phospholipid head groups shift and membrane thinning [141]. During the process of penetration into the phospholipid bilayer, the helical hydrophobic regions of the α-helical peptides and β-sheet peptides are close to the hydrophobic regions of the membrane phospholipid, while the hydrophilic regions of the peptide helixes are inwards, and multiple helical molecules are arranged in parallel to form the central lumen [137].

While the mechanism of toroidal-pore model is similar to that of barrel-stave model, the difference is that in the toroidal-pore model the peptide helixes insert into membrane and bind with lipids to form toroidal pore complexes. Locally accumulated AMPs at high concentrations induce deformation of bending in lipid molecules, thus enabling the peptides and lipid head groups embedded inside of the lipid hydrophobic center [141].

In the carpet model, while the electrostatic effect of AMPs and anionic membrane is necessary, the high AMP concentrations are required to form micelle and destroy the microbial membrane [137]. When the peptide concentration reaches the threshold, AMPs cover the membrane in clusters and cause the membrane rupture in a surfactant-like manner. Neither channel formation nor insertion of the peptides into the hydrophobic center of the membrane occurs. This effect is potent enough to induce the completely or partially cell membrane lysis with the result of cell death.

In the aggregate model, AMPs bind to the anionic cytoplasmic membrane, forcing the peptides and lipids to form a peptide-lipid complex micelle [142]. Different from the carpet model, the channels formed by AMPs, lipids and water allow ions and intracellular contents to leak out, and then lead to cell death. These channels may also help AMPs transfer into the cytoplasm and exert function. This mechanism explains why AMPs not only target the cytoplasmic membrane, but may also cross the membrane into the cytoplasm to act on intracellular substances [143].

Unlike the cationic AMPs, the mechanisms of anionic AMPs action remain elusive. The antibacterial mechanism of maximin H5 against *Staphylococcus aureus* (*S. aureus*) has been considered to be associated with the membrane dissolution [144]. The maximin H5 interacts with microorganisms through its N-terminal α-helical peptide where the aspartic acid residues only play a major structural role due to their distance to the membrane surface. Hydrogen bonds formed by amidation of C- and N-terminal is crucial for stabilizing the α-helix structure of the peptide [87]. Besides, low pH appears to help enhancing the degree of α-helix of maximin H5 and promotes to kill *S. aureus* in a “Carpet”-like mechanism [144]. The anionic AMP Xlasp-p1 exhibits a significant broad-spectrum antibacterial activity against both Gram-positive and Gram-negative bacteria by destruction of cell membranes and intracellular material efflux [145]. A recent study reported that the anionic AMP AP2 reduced the survival of *C. albicans* cells, but had no effect on the activity of protoplast, suggested AP2 may act on fungal cell walls [146].

In addition to destruction of membrane, other modes of action have also been reported. For instance, in neutral pH, clavanin A adopts the membrane permeation mode of α-helical peptide [147]. But in slightly acidic pH, it induces cell death by acting proteins on the membrane that maintain a stable pH gradient. The LPS anchored in the outer membrane of the bacterial pathogen is a crucial step for microbial surface disruption. Fiorentino et al. [148] illustrated that insertion of LPS into the bacterial surface relies on the concerted opening movements of both the β-barrel and β-taco domains of LPS transport protein. Thanatin stabilizes the β-taco domain, thereby preventing transport of LPS to the cell surface [148].

### Intracellular mode of action

A growing body of evidence suggested that AMPs had other mechanisms along with membrane penetration and pore formation (Fig. 1).

#### AMPs acting on nucleic acids

Buforin II, an AMP with 21 amino acids in length, has antibacterial activity against a wide range of bacteria [149]. It has the same sequence as the part of the histone H2A, a protein that directly interacts with nucleic acids [149]. Previous studies have shown that buforin II penetrated lipid vesicles in vitro without affecting membrane permeability and bound to DNA and RNA [149]. Another study indicated that buforin II mutants exhibited a reduced interaction with DNA and activity compared with buforin II [150]. Similarly, indolicidin penetrated bacterial membranes and inhibited DNA synthesis in the absence of bacterial cell lysis [149]. Peptide-P2, an anionic antimicrobial peptide isolated from *Xenopus laevis* skin, inhibited bacterial growth by disruption of the bacterial cell membrane, and interaction with the microbial genomic DNA [151]. In addition to directly binding to DNA and inducing DNA damage, AMPs can also indirectly inhibit the DNA replication or transcription [152–154].

#### AMPs acting on protein synthesis

PR-39, an proline and arginine-rich AMP and isolated from the small intestine of pigs, was found to penetrate the outer membrane of *E. coli* rapidly [155]. Once entry into the cytoplasm, PR-39 inhibits protein synthesis and causes the degradation of proteins required for DNA synthesis, which in turn disrupt DNA synthesis. Typically, the proline-enriched AMPs interfere with protein synthesis via binding to ribosomes [156]. For example, oncocin-type peptide inhibits mRNA translation by binding 70S ribosome export, while apidaecin-type peptide blocks the assembly of the ribosome 50S large subunit [157]. Api137, an apidaecin-derived peptide, was showed to bind *E. coli* ribosomes and trap release factor 1 (RF1) or release factor (RF2) for releasing the nascent polypeptide chain, resulting in translation termination [158]. Another study showed that the N-terminal fragments (1–25) and (1–31) of nonlytic proline-rich AMP (PrAMP) Bac5 inhibit bacterial protein synthesis by binding the tunnel of ribosome and preventing the transition from the initial stage to the elongation stage of translation [159].

#### AMPs acting on the activity of enzyme

Reports have indicated that AMPs inhibit the activity of bacterial intracellular enzyme [133]. Otvos’ group showed that a PrAMP pyrrhocoricin specifically bound bacterial heat shock protein DnaK from *E. coli* protein lysates [160]. In a follow-up study, the same group demonstrated that pyrrhocoricin inhibited the ATPase actions of DnaK [161]. A ribosomal synthesized and post-translationally modified peptide, microcin J25 was found to bind to the secondary channel of the RNA polymerase and block trigger-loop folding, which is essential for efficient catalysis by the RNA polymerase. Consequently, it inhibits RNA polymerase activity by preventing the entry of substrates through this channel [162]. Yang et al. [163] discovered that LL-37 had a dramatic antibacterial effect on *E. coli *via the inhibition of activity of palmitoyl transferase PagP, which is located in the Gram-negative bacterial cell outer membrane and repairs membrane permeability through activation of lipid A acylation. Hou et al.[164] suggest that the antimicrobial peptide NP-6 from Sichuan pepper seeds strongly inhibited the β-galactosidase activity of *E. coli* in a dose-dependent manner.

#### AMPs acting on the synthesis of cell wall

HNP1 was initially found to penetrate the outer and inner membranes of *E. coli* and suppress the synthesis of DNA, RNA and protein of bacteria [165]. Notably, inner membrane permeabilization appears to be the lethal event. The antibacterial activity of cycloserine can be inhibited by cycloserine which blocks the activity of alanine racemase and _D_-Ala-_D_-Ala ligase and consequently the synthesis of _D_-Ala-_D_-Ala dipeptide of lipid II of the peptidoglycan precursor [166]. This suggests that HNP1 kills bacteria by interacting with lipid II. Teixobactin inhibits the synthesis of cell wall by binding to a highly conserved motif of lipid II and lipid III (precursors of cell wall teichoic acid) [167]. Manabe et al*.* [168] found that D-form KLKLLLLLKLK-NH_2_ peptide enhanced the membrane permeability of *S. aureus* through specifically integrating with cell wall components (including peptidoglycan), thus having higher antibacterial activity than L-form.

#### AMPs acting on other targets

Pyrrhocoricin, drosocin and apidaecin, the short PrAMPs, interact with the heat shock protein DnaK of bacterial to exert antibacterial effects [161]. Drosocin and pyrrhocoricin inhibit chaperone-assisted protein folding via binding of DnaK. The θ-defensins are the circular AMPs produced in the leukocyte of Old World monkeys. These AMPs interact with bacterial membrane. The release of cell wall lyase further hydrolyzes the sugar chain and peptide bridge of the murein, and eventually induces *Staphylococci* lysis [169]. For example, Mel4 induced cell death of *S. aureus* by inducing the release of bacterial autolysin [170]. In addition, AMP PFR induces necroptosis by endoplasmic reticulum (ER) stress, and elevated cytoplasmic calcium and mitochondrial ROS levels [171].

Recent studies suggest that the direct coaggregation of amyloidogenic peptide and amyloids is an important antibacterial mechanism of AMP action [172]. Despite the low similarity between AMPs and amyloidogenic peptides in terms of sequences, typical secondary structures, or normal biological activity, the facts of the formation of fibrils by antimicrobial peptides and the antimicrobial activity of amyloidogenic proteins indicate a potential similarity in actions [173]. Indeed, Kurpe et al. [174] demonstrated that the amyloidogenic regions of ribosomal S1 protein from *Thermus thermophilus* can act as antibacterial peptides, interacting with the “parental” S1 protein (protein of specific bacterial species) to form fibrils that aggregate and interfere with its function. The formed protein aggregates can also suppress the intracellular transport processes, sorb chaperones and the functions of other proteins and ultimately, lead to the bacterial death [172]. Interestingly, amyloidogenic regions are predicted in about half of the AMP [172], implicating the potential significance of aggregation in AMP action.

## Activity of AMPs

### Antibacterial activity

AMPs exert antibacterial activity by membrane or non-membrane mediated action. As discussed above, the cationic AMPs have a stronger affinity with microbial pathogens due to the presence of the unique anionic components in the plasma membrane of bacteria and fungi, such as LPS of Gram-negative bacteria, lipoteichoic acid of Gram-positive bacteria and mannan of fungi. AMPs cause membrane permeation or perforation to induce the leakage of intracellular contents, or penetrate into the membrane to exert intracellular actions. The rapid killing and generic membrane and intracellular effects without targeting specific molecules/pathways prevent the development of bacterial resistance to AMPs. Therefore, it is attractive to the application of AMPs to the management of antibacterial resistance. Bacteriocins are a large class of small molecule cationic AMPs (30–60 amino acids) isolated from bacteria. According to the mechanisms of peptide synthesis, they were classified into two groups. One group is the peptides synthesized by ribosomes with relatively narrow antibacterial activity against bacteria and fungi, and the other group is the peptides synthesized by non-ribosomes with broad antibacterial activity [175]. Wang et al. [176] discovered a new short non-ribosomal AMP, albopeptide 6, in the culture broth of *Streptomyces albofaciens*, which displayed a narrow-spectrum activity against vancomycin-resistant *Enterococcus faecium*. Nisin is a member of the bacteriocins family and has high antibacterial activity against a wide range of Gram-positive bacteria and even Gram-negative bacteria [177]. Tong et al. [178] reported that penicillin or chloramphenicol combined with nisin improved antibacterial effect in *E*. *faecalis* where single antibiotic alone had no significant activity. Therefore, AMPs act as the novel therapeutic option of treating antibiotic-resistant bacteria either alone or applied in a synergistic manner. It is found that the expression of AMPs in shrimp, such as CrusI-3 and Alf-E1, are directly regulated by the forkhead box transcription factor O (FoxO) but independent of the Imd signaling pathway [179]. Notably, the long-term exposure to a low concentration of AMPs can induce the resistance [180]. Thus a high concentration of AMPs is recommended for maintaining the bactericidal activity [180].

### Antiviral activity

Besides the antibacterial activity, AMPs also have a broad-spectrum antiviral activity against the enveloped viruses. For example, bovine antimicrobial peptide-13 effectively inhibits the viral proliferation by disruption of the viral protein synthesis and the viral gene expression in transmissible gastroenteritis virus [181]. The anti-herpes simplex virus (HSV) activity of AMPs, such as protegrin and indolicidin, have been attributed to blocking the adhesion and entry of the virus by targeting the viral membrane glycoprotein [182, 183]. The inhibitory effect of LL-37 on a variety of the enveloped viruses, including human immunodeficiency virus (HIV), influenza A virus (IAV), vaccinia virus (VV), HSV, dengue virus (DENV) and Zika virus (ZIKV) [184–189], is achieved by destroying the viral membrane and inhibiting DNA replication. Additionally, LL-37 and mouse CRAMP markedly inhibit non-enveloped enterovirus 71 replication via regulating antiviral response and inhibiting viral binding [190]. LeMessurier et al. [191] have demonstrated that AMPs altered the immune response to IAV infection, thereby enhancing the protection of the host against virus. Both pa-MAP and temporin B reduce the infection of HSV1 by inhibiting the attachment of the virus [192, 193]. In addition, temporin B can also destroy the virus envelope and affect the subsequent post-infection stage. Temporin G, an analogue of temporin B, showed the ability to interact with the viral hemagglutinin protein of influenza virus and consequently block the conformational rearrangements of HA2 subunit, a process which is essential for the viral envelope fusion with intracellular endocytic vesicles and the entry into the host cells [194]. In the case of parainfluenza respiratory virus, the temporin G-mediated blocking of the late steps of viral replication impairs the extracellular release of viral particles. The human α-defensin-derived peptide HD5(1–9) is also able to prevent viral infection by inhibiting the adherence and the subsequent entry of the virus into cells [195]. Cathelicidin-derived AMP GF-17 and BMAP-18 inhibit ZIKV through directly inactivating the virus and interfering with the interferon (IFN) pathway [196]. Furthermore, other AMPs also have antiviral activities against DENV and pseudorabies virus [197, 198]. Moreover, AMPs have also been reported to fight against non-enveloped viruses. For instance, LL-37 has been shown to be against non-enveloped viruses such as adenovirus, rhinovirus and Aichi virus [192, 199, 200]. AMPs not only exert a direct antiviral effect on the viral particle and its replication cycle, but also indirectly inhibit virus growth by regulating host immune response [201, 202] as discussed below. Recent studies have reported that vitamin D can induce the production of cathelicidins and defensins to reduce the rate of virus replication, thereby reducing the risk of infection and death from influenza and coronavirus disease 2019 (COVID-19) [203].

### Antiparasitic activity

A large body of literature had focused on the role of AMPs in the activity of antibacterial and antiviral, however, there is still a paucity of reports about antiparasitic activity, particularly in vivo and in clinical settings. The diversity of parasite is very large, ranging from protozoa to worms. Parasites are an important cause of the human diseases worldwide, resulting in a significant global health burden [204, 205]. Eleven parasitic infections have been identified by the World Health Organization (WHO) as neglected tropical diseases because they threaten the health of millions of individuals and disproportionately impact impoverished individuals [205]. The most important parasitic diseases including malaria, leishmaniasis, trypanosomiasis and schistosomiasis [206, 207]. AMPs-based antiparasitic therapeutic strategies has gained the substantial interest recently. Leishmanicidal AMPs have been found in different creatures, for example, (1) halictine-2, from the venom of eusocial honeybee, showed significant anti-leishmanial activity without haemolytic activity for mouse macrophages and human erythrocytes [208]; (2) attacin, cecropin and defensin 2 from *Lutzomyia longipalpis* by Toll and Imd pathways, respond to *Leishmania infantum chagasi* infection [209]; and (3) dragomide E, a linear lipopeptide isolated from the cyanobacteria *Lyngbya majuscula* with an antileishmanial activity against *Leishmania donovani* promastigotes. In addition, LZ1, a peptide derived from snake cathelicidin, showed strong suppression of blood stage *Plasmodium falciparum* by specifically inhibiting adenosine triphosphate (ATP) production in parasite-infected erythrocyte [210]. Phylloseptin-1, from the skin secretion of *Phyllomedusa azurea*, had high antiparasitic activity and prevented the development of cross-resistance because of its unique chemical structure [211].

### Immunomodulatory activity

**Fig. 2:**
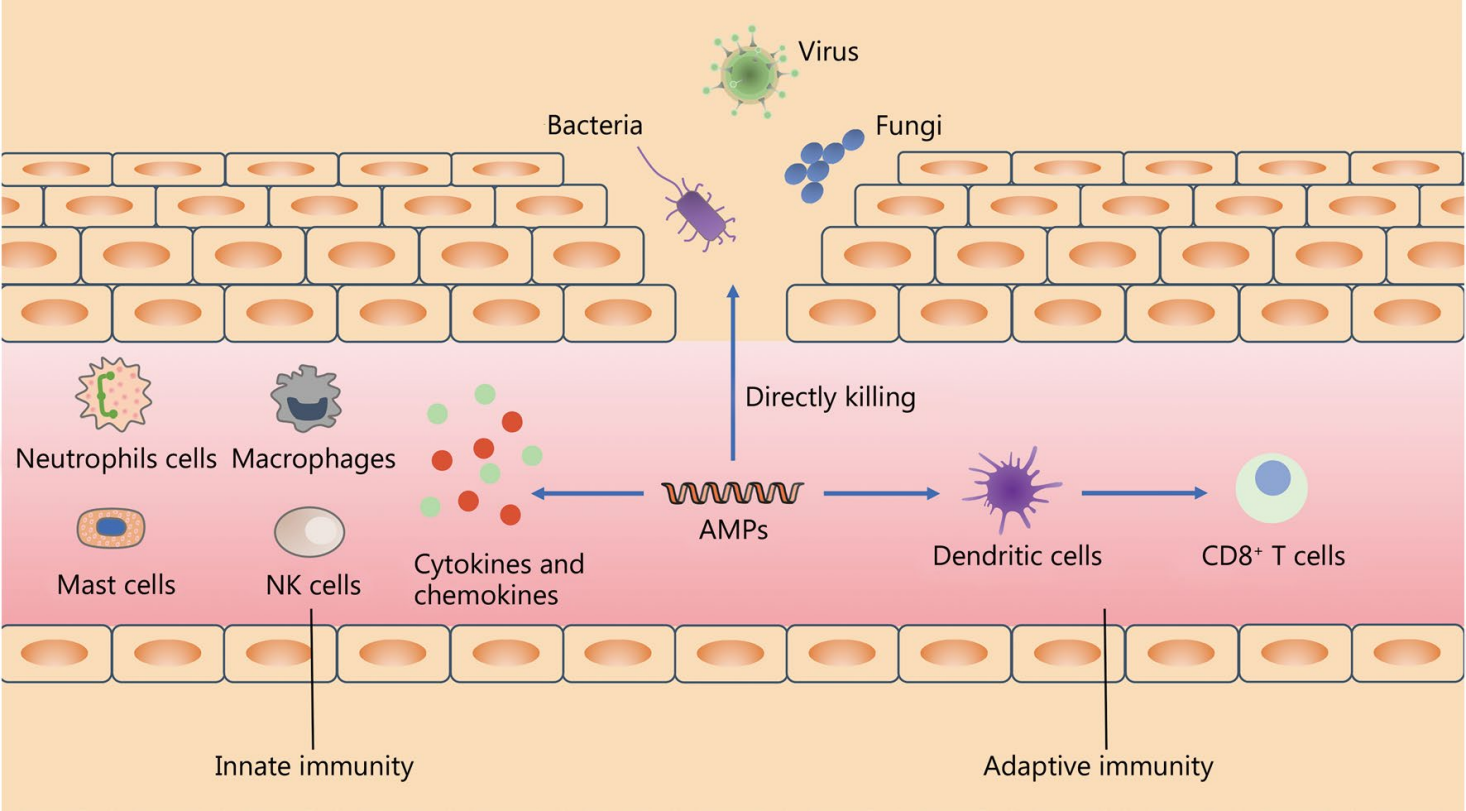
The immunomodulatory mechanisms of AMPs. AMPs not only directly kill invading pathogenic microorganisms, but also indirectly kill them by activating the immune system. On the one hand, AMPs can activate immune cells such as neutrophils, macrophages, mast cells and NK cells in the innate immune system and induce the production of cytokines and chemokines to engulf pathogenic bacteria and kill them. On the other hand, AMPs are also able to activate adaptive immune responses, present antigens to T cells by activating dendritic cells (DCs), and induce the activation of cytotoxic T cells to kill pathogenic bacteria. AMPs antimicrobial peptides; NK natural killer

AMPs, also known as the host defense peptides, protect the host from infection through antimicrobial activity and immunomodulatory effect [212–215]. The invasion of pathogens activates a series of immune responses (Fig. 2). Neutrophils are the major source of cathelicidins and defensins [6, 46]. The role of AMPs in the immune process is extremely complex. AMPs maintain the dynamic balance of the immune microenvironment through regulating the section of cytokines, such as interleukins, tumor necrosis factors (TNFs), IFNs, chemokines, and activities of immune cells such as dendritic cells (DCs), monocytes, macrophages, mast cells, granulocytes and lymphocytes. These peptides regulate the cell surface receptors such as cytokine receptors, chemokine receptors and G-protein coupled receptors (GPCRs) including formyl peptide receptors (FPRs) and Toll-like receptors (TLRs), and several intracellular signal pathways such as nuclear factor-κB (NF-κB), extracellular signal-regulated kinase 1/2 (ERK1/2), p38, JUN N-terminal kinase (JNK) mitogen-activated protein kinase (MAPK), phosphoinositide 3-kinase (PI3K).

As a component of the innate immune system, AMPs interact with immune cells to eliminate pathogens and prevent infection. LL-37 is constitutively expressed by neutrophils [66], mast cells, natural killer (NK) cells and epithelial cells [216], and recruits other immune cells to the sites of microbial invasion by binding to peptide receptor-like 1 (FPR1) (newly named FPR2) [217–219]. LL-37 also promotes the migration of mononuclear/macrophage and significantly enhances macrophage phagocytosis against Gram-positive and Gram-negative bacteria by interacting with the primary receptor integrin α_M_β_2_ (Mac-1) on the bone marrow cell surface [220]. Moreover, LL-37 induces cell chemotaxis and degranulation, and recruits mast cells to inflammatory lesions by binding to GPCR Mas-related gene X2 (MrgX2) on mast cells [220]. LL-37 is also shown to promote the formation of neutrophil extracellular traps (NETs) [216, 221] and stabilized neutrophil derived DNA or NETs to resist being degraded by bacterial nuclease. In addition, LL-37 induces activation of caspase-1, and processing and release of IL-1β through binding to P2X7 receptor in LPS-primed macrophages [222], and promotes ROS production in neutrophil [223].

LL-37 evokes the inflammatory response by stimulating immune cells to secrete chemokines and pro-inflammatory cytokines. It directly stimulates mast cells to synthesize IL-1β, IL-6, TNFs and chemokines including CCL2 and CCL3 but not CCL8 [224]. It also causes the enhancement of TLR2, TLR4 and TLR9 on the mast cell surface and TLR3, TLR5 and TLR7 in the cytoplasm, perhaps by regulating the expression of TLR to enhance the ability of mast cells to detect invading pathogens [225]. Furthermore, LL-37 promotes the expression of chemokines including CXCL8/IL-8, monocyte chemoattractant protein-1 (MCP-1) and monocyte chemoattractant protein-3 (MCP-3) by activating ERK1/2 and p38 in human blood derived monocytes, and induces chemotaxis, proliferation and differentiation of monocytes [226].

Similarly, defensins have a potent pro-inflammatory function [202]. Human α-defensins mainly consist of human neutrophil peptide (HNP) 1–4 and human defensin (HD) 5–6, and β-defensins mainly consist of HBD1-4. These AMPs are widely found in immune cells such as neutrophils, monocytes/macrophages, lymphocytes, NK cells and Paneth cells [202, 227–229]. HNP1-3 causes the release of TNF-α and IFN-γ from macrophages and acts in an autocrine manner to increase the expression of CD32 (FcγRIIB) and CD64 (FcγRI), and thereby enhance phagocytosis of macrophages [230]. HBDs regulate the activity of a wide range of immune cells, including monocytes/macrophages, DCs, memory T cells and mast cells [202, 219]. Niyonsaba et al. [231] found that HBD2-4 rather than HBD1 could stimulate the expression of IL-6, IL-10, IFN-γ-inducible protein IP-10, MCP-1, macrophage inflammatory protein-3α (MIP-3α) and RANTES/CCL5 in human keratinocytes. HBD2 and HBD3 stimulate the mononuclear and polymorphonuclear cells to produce TNF-α, IL-10 and IL-6 in an inflammatory environment, while HNP1 stimulates the mononuclear cells to produce IFN-γ, IL-10 and IL-6 [232]. Besides, these cytokines in turn promote immune cells to express more AMPs [233]. Porcine β-defensin 2 protects against bacterial infection through direct bactericide action and altered inflammation by interfering with the TLR4/NF-κB pathway and inhibiting the release of pro-inflammatory cytokines, including IL-6, TNF-α, IL-1β and IL-12 [234]. Sechet et al. [235] found that the small molecules isolated from medicinal plants, andrographolide, oridonin, and isoliquiritigenin, induced the expression of HBD3 in colon epithelial cells by targeting the epidermal growth factor receptor (EGFR)/MAPK pathway.

On the other hand, the affinity of AMPs to pathogen may facilitate infection under certain situations. By targeting human enteric HD5, *Shigella* infects intestinal epithelium through the interaction between bacterial surface proteins and HD5 to enhance adhesion and invasion of intestinal epithelium [236]. Interestingly, HD5 in macrophage can also promote the phagocytosis of *Shigella* and the bacterial replication causes macrophage cell death and the subsequent release of bacteria to infect the intestinal epithelial cells [237].

While appropriate inflammatory responses accelerate the removal of invading pathogens and infected cells, the excessive and long-term inflammation can lead to tissue damage, chronic inflammatory disease which contributes to oncogenic transformation. Therefore, when the degree of inflammation reaches a certain level, the inflammatory response should be controlled to maintain microenvironment homeostasis. AMPs have either pro-inflammatory or anti-inflammatory effects according to their expression levels in the sites of inflammation [238]. Hosoda et al*.* [239] found that LL-37 not only released NETs to inhibit the growth of bacteria, but also improved the survival of cecal ligation and puncture (CLP) sepsis mice by alleviating inflammatory responses through reducing cytokines, soluble TREM-1 and danger-associated molecular patterns (DAMPs). Furthermore, LL-37 mediates the internalization of the chemokine receptor CXCR2 in the monocytes and neutrophils and subsequently attenuates their chemotaxis [240]. LL-37 also significantly reduces the release of pro-inflammatory cytokines in LPS-stimulated neutrophils while inducing the production of intracellular ROS and the intracellular ingestion of bacteria [241]. In addition, LL-37 suppresses *Aspergillus fumigatus* infection by binding the fungal hyphae, and reduces the release of pro-inflammatory cytokines by macrophages [242]. Therefore, AMPs exert immunomodulatory effect to prevent infection-associated tissue damages and maintain microenvironment homeostasis.

### Tumor modulatory activity

**Fig. 3:**
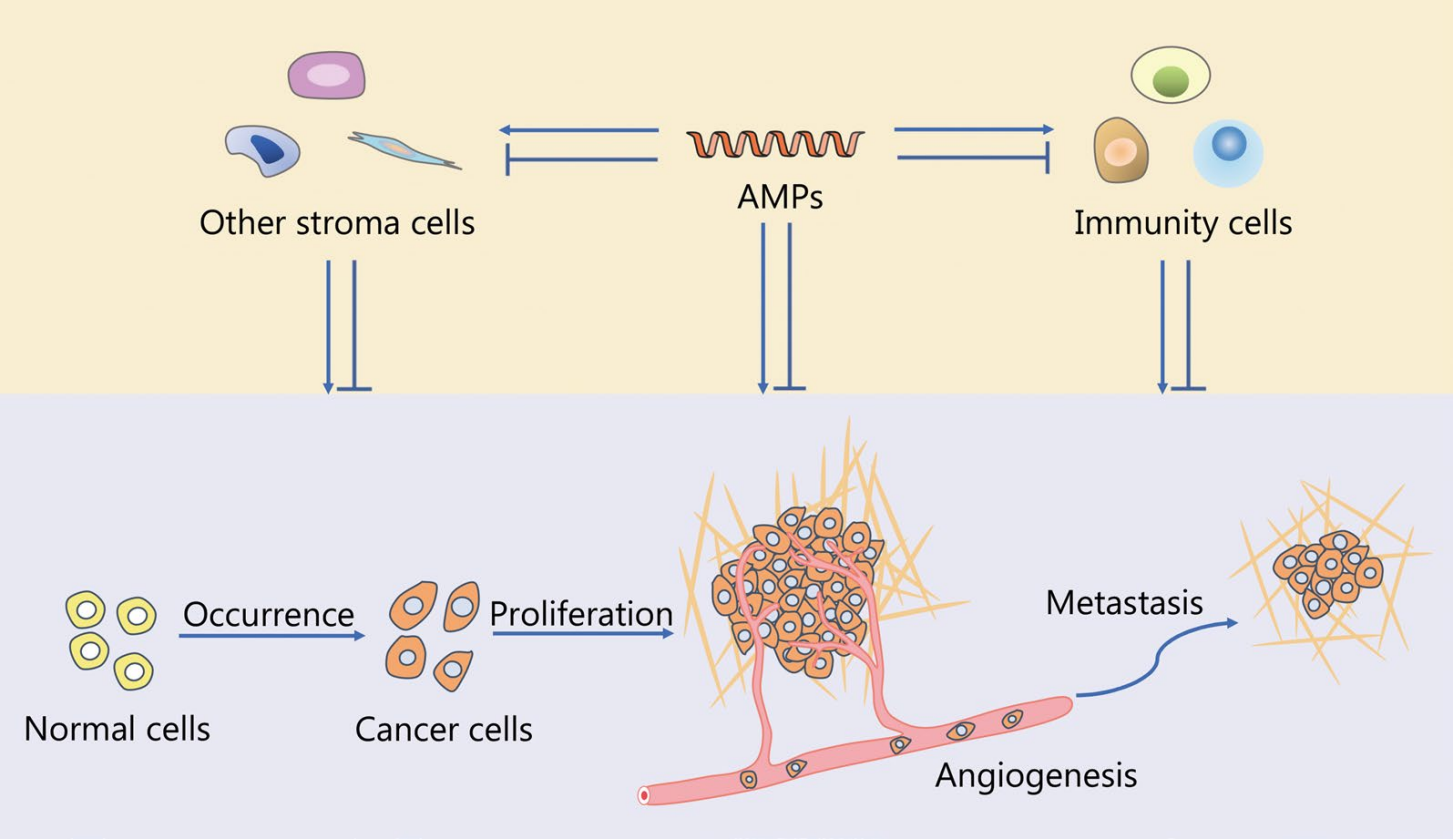
The tumor modulatory mechanisms of AMPs. AMPs play a dual role in promoting or inhibiting the occurrence and development of cancer. AMPs not merely directly affect the process of the occurrence of cancer cells, cell proliferation and metastasis, but also promote or inhibit these capabilities of cancer cells by mediating stromal cells in the immune microenvironment and other tumor microenvironment. AMPs antimicrobial peptides

Growing evidence supports an anticancer activity of AMPs [3, 243]. AMPs selectively kill cancer cells by acting on the membrane surface. Compared with normal cells, the anionic composition of cancer cells’ membrane surface confers the targeting specificity of AMPs. Paradoxically, AMPs can promote tumor progression in the certain types of cancer. Thus, the functional role of AMPs in cancer cells is tumor type specific [244] (Fig. 3).

LL-37, the only cathelicidin in humans, inhibits tumor growth in colon cancer [245–247] and gastric cancer. LL-37 induces apoptosis-inducing factor (AIF)/endonuclease G (EndoG) mediated apoptosis by activating the GPCR-p53-Bax/Bak/Bcl-2 signaling cascade in colon cancer cells. Cathelicidin-deficient mice showed a higher sensitivity to azoxymethane-induced colon cancer occurrence [248]. Cathelicidin has been reported to inhibit colon tumor growth and metastasis through P2RX7-dependent pathways in mice [249]. Hayashi et al. [250] found that FF/CAP18, an analog of LL-37, was localized to the cytoplasm of colon cancer cells and enhanced the expression of growth-suppressing miRNAs. These miRNAs were also transported to other cancer cells via exosomes to inhibit proliferation. In gastric cancer, the abundance of LL-37 is lower than in normal tissues [247]. LL-37 activates bone morphogenetic protein (BMP) signaling through a proteasome-dependent mechanism to inhibit gastric cancer cell proliferation [251]. In addition, other AMPs including KT2 [252, 253], BG-4 [254] and KL15 [255] induce apoptotic or necrotic cancer cell death.

Paradoxically, LL-37 promotes tumorigenesis in breast cancer, ovarian cancer, malignant melanoma, lung cancer, prostate cancer, pancreatic cancer and squamous cell carcinoma of the skin [256]. For instance, LL-37 was found to be highly expressed in breast cancer [257], and promoted the migration and metastasis of breast cancer cells [258]. In ovarian cancer, it enhances the proliferation, migration and invasion of ovarian cancer cells [247]. This pro-tumorigenic effect of LL-37 is associated with the immune modulation. Ovarian cancer cells produce versican V1 which induces the production of LL-37 by activating macrophage TLR2 and vitamin D_3_ signals. LL-37 promotes ovarian tumor growth by recruiting multipotent mesenchymal stromal cells [259]. LL-37 also promotes the proliferation, migration and invasion of melanoma cells by activating the NF-κB pathway [260], and promotes the growth of lung cancer through the Wnt/β-catenin and MAPK signaling pathways [261]. A recent research found that LL-37 secreted by tumors may be used as an immunosuppressive cytokine to induce tumor immune tolerance by converting effector Th17 cells into suppressor Th17 cells [262].

Like cathelicidins, defensins act as a double edge sword in the development of cancer pathogenesis. High concentrations of HNP1, HNP2 and HNP3 (HNP1-3) were found to be positively correlated with cell necrosis in renal cell carcinoma (RCC) tissues [263]. A significantly increased level of HNP1-3 was also detected in cancer tissues and serum of patients with metastatic colorectal cancer [264]. Similar to HNP1-3, a high level of HD6 was detected in colon cancer tissues and colon cancer cell lines [265], supporting a potential prognostic value of HNP1-3 and HD6 in colorectal cancer. On the other hand, the anti-tumor activity of HNP1-3 has been reported in some types of tumors. HNP1 inhibits tumor growth in lung adenocarcinoma [266], colon and breast tumors by inducing apoptosis, reducing angiogenesis and mediating anti-tumor immunity [267]. It also significantly improves the efficacy of doxorubicin in breast cancer and lactoferrin in oral squamous cell carcinoma (OSCC) [268]. Moreover, HNP1-3 derived from neutrophils have cytotoxic effects on OSCC cells [269].

As another major class of defensins in humans, low expression levels of HBD1, HBD2 and HBD3 had been reported in colon cancer [207, 270] and OSCC [271, 272], while one study showed increased expression of HBD2 in OSCC [273]. This apparent contradiction may be explained by the level of inflammation in the biopsy section [272]. The low protein expression of HBD1 has been detected in 82% of prostate cancer, 90% of RCC and liver cancer [274–276]. The oncogenic EGFR-ERK-MYC signal axis suppressed the expression of HBD1 in colon cancer [277]. Overexpression of HBD1 leads to caspase-3-mediated apoptosis in renal cancer cells SW156 and epidermoid carcinoma cells [275]. HBD1 also inhibits the growth of bladder cancer through the HER2-ERK pathway [278, 279]. Like HBD1, the low expression of HBD2 has been reported in oral tongue squamous cell carcinoma [280]. Its tumor inhibitory effect on colon tumor is through anti-tumor immunity [281]. This immune modulatory effect was further demonstrated that infection with a recombinant VV expressing HBD2 led to recruitment of the plasmacytoid DCs to the tumor sites, enhancing cytotoxic T cells to attack tumor cells, thereby inhibiting tumor growth [282]. HBD3 also inhibits the migration of head and neck cancer cells [283], and the growth of lung cancer [284]. The HBD3 produced by tumor-infiltrating neutrophils inhibited the migration of colon cancer cells through metastasis-related 1 family member 2 in a paracrine manner [207]. On the other hand, an oncogenic role of HBD3 has been suggested in cervical cancer by activating NF-κB signaling [285], and in head and neck squamous cell carcinomas (HNSCC) by inducing the expression of programmed death-ligand 1 (PD-L1) [286].

θ-defensin derivatives specifically inhibit the proliferation of breast cancer cells but spare normal breast epidermal cells [287]. Homozygous deletion of the θ-defensin gene in different cancers activates oncogenic pathways and suppresses immune response pathways [288], implicating its potential as a prognostic biomarker for immunotherapy. Treatment with the plant-derived natural defensin PvD1 in breast cancer cells inhibits tumor growth by modulating the exosomal membrane composition [289]. A novel frog skin-isolated peptide dermaseptin-PP exerts the anti-tumor activity in lung cancer cells by inducing cell apoptosis via both endogenous mitochondrial apoptosis pathway and exogenous death receptor apoptosis pathway [290]. PFR peptide induces necroptosis of acute myeloid leukemia cells by inducing endoplasmic reticulum stress and mitochondrial oxidative stress [171]. The diverse roles of other AMPs in cancer have been summarized in Additional file 1: Table S2.

### Other activities

AMPs can promote wound healing [291–293] after skin injury, a process involving the complex interactions of keratinocytes, fibroblasts, vascular endothelial cells, immune cells and the extracellular matrix [294–296]. Some AMPs play a vital role in both skin barrier and function [297] and thus have the potential to treat multiple skin maladies, exemplified by melanoma, acne, diabetic foot ulcer and psoriasis [298]. Experimental studies revealed that LL-37 [299] and S100 peptide [300] in keloid tissues mitigated collagen production, supporting the AMPs’ antifibrogenic properties. Yan et al. [301] recently reported that the anti-fibrotic properties of AMP YD were mediated through the miR-155/Casp12/NF-kB pathway. In addition, the recombinant LL-37 can induce endothelial cell proliferation, migration and formation of tubule-like structures, and increase vascularization and re-epithelialization in mouse trauma experiments [302].

AMPs are related to the occurrence and development of diabetes [303, 304]. In patients with type 2 diabetes, the level of LL-37 in serum was found to be positively correlated with inflammation markers and negatively correlated with the level of high-density lipoprotein (HDL) [305]. Under the influence of short-chain fatty acids produced by intestinal microorganisms, cathelicidin related antimicrobial peptide (CRAMP) produced by pancreatic β-cells induces activation of the regulatory immune cells and thereby reducing the incidence of autoimmune diabetes [306].

AMPs can regulate NETs and inflammation and are involved in the process of sepsis infection. LL-37 improved the survival of polybacterial septic mice by neutralizing the effects of LPS and inhibiting ATP-induced/P2X7-mediated pyroptosis of macrophage, a caspase-1 dependent cell death and inflammatory cytokine production [307, 308]. In addition, AMPs play a vital role in maintaining colon homeostasis, tissue repair and preventing cancer by maintaining the balance of colon microbiota [309–311].

## Strategies of AMPs for clinical application and development

The inappropriate and excessive use of antibiotic leads to antibiotic resistance, a major clinical challenge. AMPs with a broad-spectrum antibacterial activity are expected to become the alternative antibiotics through the development of AMPs-based therapies. Currently, three AMPs have been approved for antibacterial treatment by the Food and Drug Administration (FDA) and another three AMPs are under the clinical development (Additional file 1: Table S3).

Cancer patients are often accompanied by an inflammatory response and the risk of postoperative pathogen infection. The antibiotic resistance has a significant impact on cancer patient survival. The immunomodulatory function and the direct anti-tumor activity make AMP-based therapies as an attractive treatment option for cancer patients. Three AMPs have been tested in the clinical trials for cancer treatment (Additional file 1: Table S3). To support the value of AMP in cancer treatment, a study recently demonstrated that tumor samples contained abundant microbiome [312]. Conventional antibiotics alone may not effectively eliminate the bacteria in tumor cells. Therefore, as both the anticancer peptides and antibacterial agents, AMPs open a new perspective for the treatment of cancer.

However, development of AMP-based therapy has encountered many challenges including stability, efficacy and toxicity, which limit the clinical development of AMPs. Particularly the undesirable pharmacodynamics of AMPs including the instability of AMPs resulting from the degradation of AMPs by the presence of proteolytic enzymes in the serum [313]; the neutralization of AMPs antitumor activities by the negatively charged proteins and high/low density lipoproteins [314]; and the rapid clearance by kidney and liver [315], their therapeutic applications. Therefore, new technologies should be exploited to improve the bioavailability of AMPs.

## Rational engineering of AMPs

Novel technologies can be applied for AMP engineering to improve their stability, activity and targetability, such as isomerization, peptide lipidation, glycosylation, cyclization, other biomimetic terminal modification and multimerization [316–318]. The activity of AMPs is influenced by many factors, such as peptide length, net charge, hydrophobicity and secondary structure. The antimicrobial activity is varied by the peptide length because peptides need to span the lipid bilayer in order to stabilize the pore [319]. But with the change of the peptide length, the net positive charge and hydrophobicity are also changed. The increased AMP positive charge results in an enhanced peptide binding to the anionic bacterial membranes [320]. However, the biological effect of highly charged peptides is significantly reduced at high ionic strength [319]. In the presence of hydrophobic groups, peptide chains can form polymers in solution which enable peptides to insert into the hydrophobic membrane core. Hydrophobic residues also increase the ability of the AMPs to form α-helix and the stability. When the AMPs form a certain secondary structure, it shows obvious amphiphilicity. The amphiphilicity is an important structural basis of AMPs. However, some studies have shown that high amphiphilicity decreased the antibacterial activity of AMPs, and led to an increase of hemolytic activity [321, 322]. For these reasons, there is no standard solution to optimize AMP engineering with coordinating various factors simultaneously.

L-to-D isomerization is a common method that enhances the proteolytic stability of peptide against a range of host and microbes’ proteases. L-amino acids are easily degraded. In order to increase their stability in serum, cyclization of AMPs, the addition of unnatural amino acids and D-amino acids are often used to modify AMPs [323–325]. For instance, chicken cathelicidin-2 after D-amino acid substitution and head-to-tail cyclization, showed enhanced serum stability and reduced cytotoxicity without affecting antibacterial and LPS neutralizing activity [324]. _D-Arg-_W3R6, an analogue of AMP W3R6 after partial D-amino acid substitution, showed increasing resistance to proteolytic enzymes without changing its antibacterial activity [326]. The mammalian HBcARD peptide after D-amino acid substitution also showed better stability, stronger antibacterial activity and very low hemolytic activity [327]. The di-substituted β-amino acids within the peptide enhance the stability, lipophilicity and ability of AMPs to penetrate target cells [328]. In addition, AMPs, linker and targeting peptide can be connected by the peptide bonds to form specifically targeted antimicrobial peptides (STAMP). The linker containing L-type or D-type amino acid enantiomers increases the stability and activity of AMP or the targeting peptide [329].

Cyclization of peptides is a particularly promising approach for improving both stability and bioactivity of AMPs [330]. Cyclic peptides bind strongly to bacterial membrane by forming a β-sheet structure at the membrane surface [331]. Dathe et al. [332] designed a series of short cyclic hexapeptides that possessed a higher antimicrobial efficacy against *Bacillus subtilis* and *E. coli* than compared to the linear form. A recent study reported that the analogues of a cyclic AMP with a flexible linker exhibited improved activity against *S. aureus* and *P. aeruginosa* compared to the original linear peptide [333]. Another form of cyclization is to rely on the disulfide bond formation to create the intramolecular cross-link between cysteine residues, which enhances proteolytic stability [334].

Stapling is a key technique of forcing peptides structure into an α-helical by the linkage of the side chains [335]. A very recent report from Demizu’s group designed and synthesized magainin 2 derivatives by stapling between the first and fifth position from the N-terminus, which showed a higher antimicrobial activity against both Gram-positive and Gram-negative bacteria than magainin 2, without exerting significant hemolytic activity [336].

The combination of two AMPs was reported to produce a stronger activity against bacteria [337]. However, the issue of host toxicity remains unresolved. Later, a more attractive hybridization strategy was proposed that the new synthesized AMP involves the combination of key residues from 2 to 3 peptides of different mechanisms of actions into a single sequence [338, 339]. The group of Alzoubi designed a new hybrid peptide H4 by combining two individual α-helical fragments of both BMAP-27 and OP-145, which displayed a broad spectrum of activity and reduced the toxicity profiles [338]. In another study, the “triple hybrid” of cecropin-A, melittin, and LL-37 significantly enhanced the bactericidal against a range of Gram-positive and Gram-negative organisms and lowered hemolytic activity [340]. Antibiotics-peptide conjugates (APCs) are a combination of known antibiotics with a peptide connected through a linker. The rationale is to produce an alternative multifunctional antimicrobial compound that will elicit synergistic antibacterial activities while reducing known shortcomings of antibiotics or peptides, such as cellular penetration, serum instability, cytotoxicity, hemolysis and instability in high salt conditions [341].

AMPs have the ability to self-assemble into an ordered amyloid-like nanostructures which facilitate their antibacterial activity by achieving more specific and stronger interactions with microbial membranes [342]. Nanomaterials can effectively kill bacteria by destroying bacterial cell membrane and causing intracellular material leakage. During membrane penetration, nanomaterials can bind to many components in bacterial cells, such as DNA, ribosomes and enzymes, and disrupt normal physiological activities of the cell, resulting in the oxidative stress, electrolyte imbalance, enzyme inhibition and other bacteriostatic effects, and ultimately lead to cell death [343].

## Delivery system

Some AMPs not only inhibit the growth of tumor cells, but also have cytotoxicity to normal cells [314]. This non-specificity is a major obstacle for successful AMP-based therapy [344]. In order to achieve tumor specific targeting, vector-mediated gene delivery AMPs has been proposed.

The nanotechnology provides stability and controlled release of AMPs to increase target selectivity. Nanostructure can improve pharmacodynamics of AMPs by inhibiting renal clearance and enhancing retention and permeability [345]. Some nanomaterials not only can enhance the stability and activity of AMPs but also have antibacterial effects [346]. Nano-delivery systems can optimize the pharmacokinetics and biodistribution of AMP, and improve biosafety and antibacterial effectiveness [347]. The types of nanostructures used in AMP delivery systems include metal nanoparticles, carbon nanotubes, lipid-based nanoparticles and polymer-based nanostructures [345]. Lam et al. [348] synthesized structurally nanoengineered antimicrobial peptide polymers (SNAPPs) by α-amino acid N-carboxyanhydrides (NCAs)-ring-opening polymerization (ROP), and exhibited sub-μM activity against all Gram-negative bacteria tested, and demonstrated low toxicity. It was reported that a new nanosystem that the encapsulation of SET-M33 peptide in single-chain dextran nanoparticles markedly inhibited *P. aeruginosa* infections [349]. In addition, generation of AMP-magnetic nanoparticles has been proposed to increase target specificity by immobilizing AMP on the surface of magnetic nanoparticles and applying an external magnetic field to control its delivery [350]. A new class of three-dimensional nanostructures, tetrahedral framework nucleic acids (tFNAs), also possesses a desirable cell-entry performance and has been utilized as a delivery vehicle [351]. In recent years, a significant progress has been achieved in the field of using nanosystems to transform or deliver AMPs, making AMPs truly an effective substitute for antibiotic therapy [347, 352–355]. However, as drug delivery systems, there are still several key issues around the drug delivery systems such as biocompatibility and nanoparticles deposition [356, 357].

Cell-penetrating peptides (CPPs) are efficient vehicles that can deliver various cargos across the biological membranes to maximize their intracellular activities [358]. Thus, fusing AMPs with CPPs could be a simple and feasible method to improve the bioactivity of AMPs. Accumulating evidences support the generation of cell-penetrating antimicrobial peptides as a new perspective for targeting intracellular infections [359]. As Lee et al*.* [360] elegantly showed conjugated CPP (R9) to AMPs (magainin and M15) significantly enhanced antimicrobial activity against Gram-negative bacteria, probably due to an increased efficiency of translocating across a lipid bilayer. Another example of CPP-AMP conjugation was reported by Hoffmann’s group through coupling the PrAMPs with penetratin (residues 43 to 58 in the antennapedia homeodomain) via their C-terminally adding cysteine to shut into mammalian cells [361]. Both AMPs and CPPs are membrane-active peptides because of the similar action on membranes and the common physicochemical characteristics. The latarcin derived peptide (LDP)-nuclear localization sequence (NLS) derived CPPs is a dual action peptide with AMP and CPP activity [362]. Drexelius et al. [363] recently reported the optimization of a CPPs C18 towards its antimicrobial activity. Surprisingly, the peptide has not only antibacterial activity but also specific antitumor activity. Therefore, connection of peptides with CPPs can increase the therapeutic efficacy and specificity of AMPs in cancer treatment. Hao et al. [364] used the TAT protein of the HIV virus as a CPP, then combined this CPP with the amphiphilic α-helical anti-cancer peptide (ACP). The CPP-ACP complex showed a potent inhibitory effect on the growth of cancer cells, and reduced the toxicity on human erythrocytes.

## Drug combination

Antibiotics in combination with AMPs is a potential therapeutic approach to overcome the antibiotic resistance, improve the killing effect and reduce concentration-associated toxicity or side effects of antibiotics. This strategy can increase the bacterial membrane permeability, decrease the efflux of antibiotic agents, affect intracellular ion homeostasis, and thus, inhibit biofilm formation and bacterial survival [365].

Several AMPs, HsAFP1, RsAFP2 and RsAFP1, showed a synergistic activity with the antimicrobial agents in treating both plankton and biofilm cells [366]. Nisin combined with the antibiotics, such as penicillin, chloramphenicol, ciprofloxacin, indolicidin, or azithromycin, showed the synergistic effect on methicillin-resistant *S. aureus* by preventing biofilm formation or inhibiting attachment of bacteria to solid surface [367, 368]. Li et al*.* [369] demonstrated that combination of tetracycline antibiotics demeclocycline hydrochloride (DMCT) and the antimicrobial peptide SAAP-148 has a synergistic antibacterial activity to combat multidrug-resistant (MDR) *P. aeruginosa* strains PAO1 and *P. aeruginosa* ATCC27853. The liver-expressed antimicrobial peptide 2 (LEAP-2), which derived from fish innate immune system, increased the activity of ampicillin against *Vibrio parahaemolyticus*, thus overcoming ampicillin-resistant *Aeromonas hydrophila* infection [370]. In addition to LL-37 from humans [371], *Xenopus laevis* antibacterial peptide-P2 from *Xenopus laevis* [151] and HsAFP1 from plant [372] in combination with the antibiotics effectively control bacterial or fungal infection. Casciaro et al. [373] also found that esculentin-1a derived antipseudomonal peptides from frog-skin had the ability to improve the activity of aztreonam in inhibiting growth and killing pseudomonas cells.

A combination of two or more types of AMPs can also achieve better efficacy. However, only a few examples of synergistic AMPs have been reported [374], including PGLa and magainin 2, which are two amphiphilic α-helical membranolytic peptides from frog skin and belong to the magainin family [375]. The team of Ulrichy proposed a new molecular model for the functionally active PGLa-magainin 2 complex in which each PGLa monomer bound to one magainin 2 molecule at its C-terminus [376]. Ma et al. [377] found that while PGLa inserted into and extracted from a membrane rapidly whereas magainin 2 tended to aggregate on the membrane surface, formation of the PGLa-magainin 2 heterodimers enabled the PGLa and MAG2 residues to be well integrated into the membrane. The combination of magainin 2 and tachyplesin 1 also enhances the bacterial membrane recognition by constituting the oligomeric structures before contacting the anionic bacterial membrane surface [378]. In addition, a recent study from Bhunia’s group reported that two AMPs, VG16KRKP and KYE28, exhibited synergistic antimicrobial effects against plant pathogens and proteases through formatting an unusual peptide complex [379].

The synergistic antimicrobial effects can also be achieved by combining AMPs with other compounds or drugs. A better antibacterial activity of nisin combined with citric acid against *S. aureus* and *L. monocytogenes* resulted from a stronger damage to the cell morphology and greater release of cell constituents [380]. Ahn et al. [381] showed that the C-terminal 15 amino acids of HBD3-C15 potentiated the bactericidal and anti-biofilm activity of disinfectants used in dental clinics against *Streptococcus mutans*, as well as calcium hydroxide and chlorhexidine digluconate. The treatment with nisin A and epsilon-poly-L-lysine showed a synergistic activity against Gram-positive food-borne pathogens *Bacillus cereus* and *L. monocytogenes* [382]. Notably, the development of the synthetic antimicrobial polymers driven by the advance of controlled polymerization techniques and the desire to mimic AMPs is an innovative approach to combating the increasing prevalence of MDR infections [383]. The bactericidal activity can be further increased by the different combination therapies involving synthetic antimicrobial polymers [348, 383–385].

## Conclusion and outlook

Taken together, AMPs have a broad-spectrum anti-pathogenic activity, as well as powerful immune regulation and anti-cancer properties. AMPs have a strong cell killing effect on MDR bacteria and cancer cells. Therefore, AMPs offer a promising revenue to address the problem of antibiotic resistance and chemotherapy resistance of cancer cells. On the other hand, their shortcomings, such as poor stability, toxicity and other side effects, may limit the clinical development. The emergence of new technologies allows us to transform the natural AMPs and synthesize new AMPs by effectively exploiting the desirable characteristics, such as amphiphilicity and lipophilicity. Furthermore, the combination strategies with AMPs holds the potential to reduce the toxicity and side effects and prevent drug resistance. Although a few AMPs have already been approved by the FDA or are in the late stages of clinical trials, the exploration road ahead is still long. We are looking forward to developing the AMP-based treatment strategies with improved safety, specificity and efficacy for bacterial infection and cancer therapy in the future.

## Supplementary Information

**Additional file 1**. **Table S1**. Structure and characteristics of AMPs. **Table S2**. The mechanism of anti-cancer activity of AMPs. **Table S3**. Selected AMPs in clinical phase of development.

## Data Availability

Not applicable.

## Abbreviations

ACP: Anti-cancer peptide
APCs: Antibiotics-peptide conjugates
AIF: Apoptosis-inducing factor
AMPs: Antimicrobial peptides
ATCUN: Amino terminal copper and nickel
BMP: Bone morphogenetic protein
*C. albicans*: *Candida albicans*
CL: Cardiolipin
CLP: Cecal ligation and puncture
CpG-ODN: CpG oligodeoxynucleotides
CPPs: Cell-penetrating peptides
CRAMP: Cathelicidin related antimicrobial peptide
DAMPs: Danger-associated molecular patterns
DBAASP: Database of Antimicrobial Activity and Structure of Peptides
DCs: Dendritic cells
DENV: Dengue virus
DMCT: Demeclocycline hydrochloride
DPG: Diphosphatidylglycerol
EGFR: Epidermal growth factor receptor
*E. coli*: *Escherichia coli*
*E. faecalis*: *Enterococcus faecalis*
EMT: Epithelial-mesenchymal transition
EndoG: Endonuclease G
ERK1/2: Extracellular signal-regulated kinase 1/2
FDA: Food and Drug Administration
FoxO: Forkhead box transcription factor O
FPRs: Formyl peptide receptors
GPCR: G-protein coupled receptor
HBD: Human β-defensin
HD: Human defensin
HDL: High-density lipoprotein
HIV: Human immunodeficiency virus
HLH: Helix-loop-helix
HNP: Human neutrophil peptide
HNSCC: Head and neck squamous cell carcinomas
HSV: Herpes simplex virus
IAV: Influenza A virus
IFNs: Interferons
JNK: JUN N-terminal kinase
LEAP-2: Liver-expressed antimicrobial peptide 2
LDP: Latarcin derived peptide
*L. monocytogenes*: *Listeria monocytogenes*
LPS: Lipopolysaccharide
MAPK: Mitogen-activated protein kinase
MCP-1: Monocyte chemoattractant protein-1
MDR: Multidrug-resistant
MIC: Minimal inhibitory concentration
MIP-3α: Macrophage inflammatory protein-3α
MrgX2: Mas-related gene X2
NCAs: N-carboxyanhydrides
NETs: Neutrophil extracellular traps
NF-κB: Nuclear factor-κB
NKs: Natural killer cells
NLS: Nuclear localization sequence
OSCC: Oral squamous cell carcinoma
*P. aeruginosa*: *Pseudomonas aeruginosa*
PC: Phosphatidylcholine
PD-L1: Programmed death-ligand 1
PE: Phosphatidyl ethanolamine
PG: Phosphatidylglycerol
PI: Phosphatidylinositol
PI3K: Phosphoinositide 3-kinase
PrAMPs: Proline-rich AMPs
PS: Phospholipids such as phosphatidylserine
RCC: Renal cell carcinoma
RF: Release factor
ROP: Ring-opening polymerization
ROS: Reactive oxygen species
*S. aureus*: *Staphylococcus aureus*
SAAP: Surfactant associated anion peptide
SDS: Sodium dodecyl sulfate
SNAPPs: Structurally nanoengineered antimicrobial peptide polymers
STAMP: Specifically targeted antimicrobial peptide
TAFs: Tumor-associated fibroblasts
TBD-1: Turtle β-defensin 1
tFNAs: Tetrahedral framework nucleic acids
TLRs: Toll-like receptors
TNFs: Tumor necrosis factors
VV: Vaccinia virus
ZIKV: Zika virus
