# Supplementary material for: Antimicrobial peptides: mechanism of action, activity and clinical potential
Source: Mil Med Res. 2021 Sep 9;8:48. doi: 10.1186/s40779-021-00343-2 (PMC8425997; doi:10.1186/s40779-021-00343-2)
Supplement: Supplementary file 1 — Additional file 1. Table S1. Structure and characteristics of AMPs. Table S2. The mechanism of anti-cancer activity of AMPs. Table S3. Selected AMPs in clinical phase of development. [file 40779_2021_343_MOESM1_ESM.docx]

**Table S1** Structure and characteristics of AMPs

| **AMP name** | **Amino acid sequence** | **Charge** | **Structure** | **Source** | **Reference** |
| --- | --- | --- | --- | --- | --- |
| **Anion AMPs** | | | | | |
| Thuricin CD | GNAACVIGCIGSCVIS**E**GIGSLVGTAFTLG | -1 | Helix | *Bacillus thuringiensis* | [1] |
| Microcin J25 | GGAGHVP**E**YFVGIGTPISFYG | -1 | β-sheet | Bacteria | [2] |
| Tricyclic peptide RP 71955 | CLGIGSCN**D**FAGCGYAVVCFW | -1 | β-sheet | Bacteria | [3] |
| Kalata B10 | GLPTCG**E**TCFGGTCNTPGCSCSSWPICTR**D** | -1 | Bridge | Plant | [4] |
| *Pv*D1 | KTC**E**NLA**D**TYKGPCFTTGSC**D** | -1 | Bridge | Plant | [5] |
| Palicourein | G**D**PTFCG**E**TCRVIPVCTYSAALGCTC**DD**RS**D**GLCKRN | -1 | Helix and  β-sheet | Plant | [6] |
| ADP-2 | YENPYGCPT**DE**GKCF**D**RCN**D**S**E**F**E**GGYCGGSYRATCVCYRT | -3 | Bridge | *Amblyomma hebraeum* | [7] |
| Bb-AMP4 | PSCVCSGF**E**TSGIHFC | -1 | Unknown | Snail | [8] |
| Maximin H5 | ILGPVLGLVS**D**TL**DD**VLGIL | -2 | Unknown | Frog | [9] |
| Chromacin | YPGPQAK**ED**S**E**GPSQGPASR**E**K | -1 | Unknown | Bovine | [10] |
| Enkelytin | FA**E**PLPS**EEE**G**E**SYSK**E**PP**E**M**E**KRYGGFM | -5 | Unknown | Bovine | [11] |
| DCD-1 | SSLL**E**KGL**D**GAKKAVGGLGKLGK**D**AV**ED**L**E**SVGKGAVH**D**VK**D**VL**D**SV | -2 | Helix | Human | [12] |
| Beta-amyloid peptide (1-40) | **D**A**E**FRH**D**SGY**E**VHHQKLVFFA**ED**VGSNKGAIIGLMVGGVVIA | -3 | Helix | Human | [13] |
| Beta2-microglobulin | IQRTPKIQVYSRHPA**E**NGKSNFLNCYVSGFHPS**D**I**E**V**D**LLKNG**E**RI**E**KVEHS**D**LSFSK**D**WSFYLLYYT**E**FTPTEK**DE**YACRVNHVTLSQPKIVKW**D**R**D**M | -2 | β-sheet | Human | [14] |
| **Cation α-helix AMPs** | | | | | |
| Cecropin A | **K**W**K**LF**KK**IE**K**VGQNI**R**DGII**K**AGPAVAVVGQATQIA**K** | +7 | Helix | Insect | [15] |
| Ceratotoxin A | SIGSAL**KK**ALPVA**KK**IG**K**IALPIA**K**AALP | +6 | Helix | Fly | [16] |
| Crabrolin | FLPLIL**RK**IVTAL | +3 | Helix | Hornet | [17] |
| Melittin | GIGAVL**K**VLTTGLPALISWI**KRKR**QQ | +6 | Helix | Honeybee | [18] |
| Pleurocidin | GWGSFF**KK**AA**H**VG**KH**VG**K**AALT**H**YL | +4 | Helix | Fish | [19] |
| Chrysophsin-1 | FFGWLI**K**GAI**H**AG**K**AI**H**GLI**HRRRH** | +6 | Helix | Fish | [20] |
| Buforin II | T**R**SS**R**AGLQFPVG**R**V**HR**LL**RK** | +6 | Helix | Frog | [21] |
| Temporin-PTa | FFGSVL**K**LIP**K**IL | +6 | Helix | Frog | [22] |
| Magainin II | GIG**K**FLHSA**KK**FG**K**AFVGEIMNS | +3 | Helix | Frog | [23] |
| Figainin 2 | FLGAIL**K**IG**H**ALA**K**TVLPMVTNAF**K**P**K**Q | +5 | Helix | Frog | [24] |
| mCRAMP | GLL**RK**GGE**K**IGE**K**L**KK**IGQ**K**I**K**NFFQ**K**LVPQPEQ | +6 | Helix | Mouse | [25] |
| ModoCath1 | VK**R**T**KR**GA**RR**GLT**K**VL**KK**IFGSIV**KK**AVS**K**GV | +12 | Helix | Mouse | [26] |
| CAP18 | GLRK**R**L**R**KF**R**N**K**I**K**E**K**L**KK**IGQ**K**IQGFVP**K**LAP**R**TDY | +12 | Helix | Rabbit | [27] |
| SMAP-29 | **R**GL**RR**LG**RK**IA**H**GV**KK**YGPTVL**R**II**R**IAG | +9 | Helix | Sheep | [28] |
| BMAP-27 | G**R**FK**R**F**RKK**F**KK**LF**KK**LSPVIPLL**H**L | +10 | Helix | Bovine | [29] |
| Cecropin P1 | SWLS**K**TA**KK**LENSA**KKR**ISEGIAIAIQGGP**R** | +5 | Helix | Pig | [30] |
| Human calcitermin | VAIAL**K**AA**H**Y**H**T**HK**E | +1 | Helix | Human | [31] |
| LL-37 | LLGDFF**RK**ARE**K**IGEEF**KR**IVQ**R**I**K**DFL**R**NLVP**R**TES | +4 | Helix | Human | [32] |
| **Cation β-sheet AMPs** | | | | | |
| Gramicidin S | VKLFPVKLFP | +2 | β-sheet | Bacteria | [33] |
| Lactococcin 972 | EGTWQHGYGVSSAYSNYHHGSKTHSATVVNNNTGRQGKDTQRAGVWAKATVGRNLTEKASFYYNFW | +4 | β-sheet | *Lactococcus lactis* | [34] |
| Plectasin | GFG**C**NGPWDEDDMQ**C**HNH**C**KSIKGYKGGY**C**AKGGFV**C**K**C**Y | +1 | Helix and  β-sheet | Fungi | [35] |
| Drosomycin | D**C**LSGRYKGP**C**AVWDNET**C**RRV**C**KEEGRSSGH**C**SPSLKCW**C**EG**C** | +1 | Helix and  β-sheet | Fruitfly | [36] |
| Tachyplesin I | KW**C**FRV**C**YRGI**C**YRR**C**R | +7 | β-sheet | Horseshoe crab | [37] |
| Hepcidin | **C**RF**CC**R**CC**PRMRG**C**GL**CC**RF | +5 | Bridge | Fish | [38] |
| AvBD2 | LF**C**KGGS**C**HFGG**C**PSHLIKVGS**C**FGFRS**CC**KWPWNA | +4 | β-sheet | Chicken | [39] |
| THP-2 | LF**C**KRGT**C**HFGR**C**PSHLIKVGS**C**FGFRS**CC**KWPWDA | +5 | Bridge | Bird | [40] |
| mBD-7 | NSKRA**C**YREGGE**C**LQR**C**IGLFHKIGT**C**NFRFK**CC**KFQ | +6 | Helix and  β-sheet | Mouse | [41] |
| Protegrin 1 | RGGRL**C**Y**C**RRRF**C**V**C**VGR | +7 | β-sheet | Pig | [42] |
| RTD-2 | GV**C**R**C**L**C**RRGV**C**RCL**C**RR | +6 | Bridge | Monkey | [43] |
| HNP1 | A**C**Y**C**RIPA**C**IAGERRYGT**C**IYQGRLWAF**CC** | +3 | β-sheet | Human | [44] |
| HBD1 | DHYN**C**VSSGGQ**C**LYSA**C**PIFTKIQGT**C**YRGKAK**CC**K | +4 | Helix and  β-sheet | Human | [45] |
| HD5 | AT**C**Y**C**RTGR**C**ATRESLSGV**C**EISGRLYRL**CC**R | +4 | β-sheet | Human | [46] |
| RNase 3 | RPPQFTRAQWFAIQHISLNPPR**C**TIAMRAINNYRWR**C**KNQNTFLRTTFANVVNV**C**GNQSIR**C**PHNRTLNN**C**HRSRFRVPLLH**C**DLINPGAQNISNCTYADRPGRRFYVVA**C**DNRDPRDSPRYPVVPVHLDTTI | +13 | Helix and  β-sheet | Human | [47] |
| Human drosomycin-like defensin | **C**LAGRLDKQ**C**T**C**RRSQPSRRSGHEVGRPSPH**C**GPSRQ**C**G**C**HMD | +5 | Bridge | Human | [48] |
| **Cationic AMPs** **containing special amino acids** | | | | | |
| Citrocin | **GG**V**G**KIIEYFI**GGG**V**G**RY**G** | +1 | β-sheet | Bacteria | [49] |
| SM-985 | GAGIGPGH**RR**TW**RR**WP**RRR**W**R** | +9 | Rich | Plant | [50] |
| Alloferon 2 | GVSG**H**GQ**H**GV**H**G | +3 | Unknown | Fly | [51] |
| Pyrrhocoricin | VDKGSYL**P**R**P**T**PP**R**P**IYNRN | +3 | Non-helix and β-sheet | Insect | [52] |
| Spgly-AMP | AIPAVDPF**G**RVKRSPWH**GG**TW**G**CKPIWACQNSPPYL**G** | +4 | Non-helix and β-sheet | Crab | [53] |
| Serrulin | **G**F**GGG**R**GG**F**GGG**R**GG**F**GGGG**I**GGGG**F**GGG**Y**GGG**KIK**G** | +4 | Rich | Scorpion | [54] |
| PcAst-1a | SNGYR**P**AYR**P**AYR**P**SYR**P**GK | +5 | Rich | Crawfish | [55] |
| Piscidin-1 | **FFHH**I**F**RGIV**H**VGKTI**H**RLVTG | +7 | Rich | Fish | [56] |
| Vipericidin | T**R**S**R**W**RR**FI**R**GAG**R**FA**RR**YGW**R**IALGLVG | +9 | Unknown | Snake | [57] |
| Tur1A | **RR**I**R**F**RPP**YL**PRP**G**RRPR**F**PPP**FPI**PR**I**PR**I**P** | +10 | Rich | Dolphin | [58] |
| Hg-CATH | **RR**F**RR**TVGLS**K**FF**RK**A**RKK**LG**K**GLQ**K**I**K**NVL**RK**YLP**R**PQYAYA | +16 | Unknown | Rat | [59] |
| PR-39 | **RRRPRPP**YL**PRPRPPP**FF**PP**  **R**L**PPR**I**PP**GF**PPR**F**PPR**F**P** | +11 | Rich | Pig | [60] |
| Prophenin-1 | AF**PPP**NV**P**G**P**RF**PPP**NF**P**G**P**RF**PPP**NF**P**GPRF**PPP**NF**P**G**P**RF**PPP**NF**P**G**PP**F**PPP**IF**P**G**P**WF**PPPPP**FR**PPP**FG**PPR**F**P** | +7 | Rich | Pig | [61] |
| Indolicidin | ILP**WKW**P**WW**P**W**RR | +13 | Non-helix and β-sheet | Bovine | [62] |
| Histatins 1 | DS**H**E**K**R**HH**GYRRKF**H**EK**HH**S**H**REFPFYGDYGSNYLYDN | +8 | Rich | Human | [63] |
| **Peptide fragments of antimicrobial proteins** | | | | | |
| Nemuri | DA**R**A**RR**IV**R**AG**RRR**GG**RR**GG**RR**GG**RR**SA**RK**S | +14 | Rich | Drosophila | [64] |
| Mytichitin-CB | TV**KC**GMNG**K**MP**CKH**GAFYTDT**C**D**K**NVFY**RC**VWG**R**PV**KKHC**G**R**GLVWNP**R**GF**C**DYA | +7 | Bridge | Mollusca | [65] |
| Histone | A**ER**VGAGAPVYL | 0 | Unknown | Fish | [66] |
| Cathelicidin-DM | SS**RRK**PC**K**GWLC**K**L**K**L**R**GGYTLIGSATNLN**R**PTYV**R**A | +9 | Unknown | Toad | [67] |
| dCATH | **KR**FWQLVPLAI**K**IY**R**AW**KRR** | +7 | Helix | Bird | [68] |
| Hemoglobin | FLSFPTT**K**TYFP**H**F**D**LS**H**GSAQV**K**G**H**GA**K** | +2 | Unknown | Bovine | [69] |
| Lactoferricin | G**RRRR**SVQW**C**AVSQPEAT**KC**FQWQ**R**NM**RK**V**R**GPPVS**C**I**KR**DSPIQ**C**IQA | +10 | Helix | Human | [70] |
| Thrombin | NLPIV**ER**PV**CKD**ST**R**I**R**ITDNMF**C**AGY**K**P**DE**GK**R**G**D**A**CE**G**D**SGGPFVM**K**SPFNN**R**WYQMGIVSWG**E**G**C**D**RD**G**K**YGFYTHVF**R**L**KK**WIQ**K**VI**D**QFG**E** | +2 | Unknown | Human | [71] |
| Cp1 | L**R**L**KK**Y**K**VPQL | +4 | Unknown | Bovine | [72] |
| Cateslytin | **R**SM**R**LSF**R**A**R**GYGF**R** | +5 | Unknown | Human | [73] |
| sfTSLP | MFAM**K**T**K**AALAIWCPGYSETQINATQAM**KKRRKRK**VTTN**K**CLEQVSQLQGLW**RR**FN**R**PLL**K**QQ | +12 | Unknown | Human | [74] |

Amino acid sequences are given in one-letter code. Boldface indicates anion amino acid residues, cationic amino acid residues, cysteine residues and special amino acid residues. *AMPs* antimicrobial peptides; *PvD1* *Phaseolus vulgaris* defensin D1; *ADP-2* amblyomma defensin peptide - 2; *Bb-AMP4* *Bellamya bengalensis*-antimicrobial peptide 4; *DCD-1* Dermcidin-1; *mCRAMP* cathelin-related antimicrobial peptide; *CAP18* 18-ku cationic antimicrobial protein; *SMAP-29* sheep myeloid antimicrobial peptide-29; *BMAP-27* bovine myeloid antimicrobial peptide-27; *AvBD2* avian beta-defensin 2; *THP-2* Turkey heterophil peptide-2; *RTD-2* rhesus theta-defensin-2; *HNP1* human neutrophil peptide 1; *HBD1* human β-defensin 1; *HD5* human defensin-5; *Hg-CATH* *Heterocephalus glaber* cathelicidin; *PR-39* proline/arginine-rich peptide-39; *dCATH* duck cathelicidin; *sfTSLP* long form thymic stromal lymphopoietin

**Table S2** The mechanism of anti-cancer activity of AMPs

| **AMP name** | **Amino acid sequence** | **Source** | **Cancer** | **Reference** |
| --- | --- | --- | --- | --- |
| **Antiproliferative** | | | | |
| Pardaxin | GFFALIPKIISSPLFKTLLSAVGSALSSSGGQE | Fish | Lung | [75] |
| BMAP-28 | GGLRSLGRKILRAWKKYGPIIVPIIRIG | Bovine | Breast | [76] |
| CGA-N46 | PMPVSQECFETLRGHERILSILRHQNLLKELQDLALQGAKERAHQQ | Human | Lung | [77] |
| Crotalicidin | KRFKKFFKKVKKSVKKRLKKIFKKPMVIGVTIPF | Human | Leukemia | [78] |
| Ranatuerin-2PLx | GIMDTVKNAAKNLAGQLLDKLKCSITAC | Frog | Prostate | [79] |
| TP4 | FIHHIIGGLFSAGKAIHRLIRRRRR | Fish | Lung | [80] |
| Magainin II | GIGKFLHSAKKFGKAFVGEIMNS | Frog | Bladder | [81] |
| Dermaseptin-PT9 | GLWSKIKDAAKTAGKAALGFVNEMV | Frog | Brain | [82] |
| Laterosporulin10 | ACVNQCPDAIDRFIVKDKGCHGVEKKYYKQVYVACMNGQHLYCRTEWGGPCQL | Bacteria | Solid | [83] |
| PaDef | CETPSKHFNGLCIRSSNCASVCHGEHFTDGRCQGVRRRCMCLKPC | Plant | Leukemia | [84] |
| Gramicidin A | VGALAVVVWLWLWLW | Bacteria | Bile duct | [85] |
| Turgencin A | GPKTKAACKMACKLATCGKKPGGWKCKLCELGCDAV | Sea squirt | Melanoma | [86] |
| Temporin A | FLPLIGRVLSGIL | Frog | Lung | [87] |
| P18 | KWKLFKKIPKFLHLAKKF | Synthetic | Leukemia | [88] |
| Gomesin | QCRRLCYKQRCVTYCRGR | Spiders | Melanoma | [89] |
| **Antimetastatic** | | | | |
| PR-39 | RRRPRPPYLPRPRPPPFFPPRLPPRIPPGFPPRFPPRFP | Pig | Liver | [90] |
| Nisin A | ITSISLCTPGCKTGALMGCNMKTATCHCSIHVSK | *Streptococcus lactis* | Colon | [91] |
| CecropinXJ | RWKIFKKIEKMGRNIRDGIVKAGPAIEVLGSAKAIGK | Silkworm | Esophageal | [92] |
| *Pv*D1 | KTCENLADTYKGPCFTTGSCD | Plant | Breast | [93] |
| Cecropin A | RWKLFKKIEKVGRNVRDGLIKAGPAIAVIGQAKSL | Silkworm | Esophageal | [94] |
| TP3 | FIHHIIGGLFSVGKHIHSLIHGH | Fish | Brain | [95] |
| LTX-302 | FKCRRWQWRMKKLGAPSITCVRRAF | Bovine | lymph | [96] |
| HBD2 | GIGDPVTCLKSGAICHPVFCPRRYKQIGTCGLPGTKCCKKP | Human | Colon | [97] |

*AMPs* antimicrobial peptides; *BMAP-28* bovine myeloid antimicrobial peptide-28; *CGA-N46* chromogranin A-N46; *TP4* tilapia piscidin 4; *PR-39* proline/arginine-rich peptide-39; *PvD1* *Phaseolus vulgaris* defensin D1; *TP3* tilapia piscidin 3; *LTX-302* a chemically synthesized 9-mer peptide; *HBD2* human β defensin 2

**Table S3** Selected AMPs in clinical phase of development

| **Peptide name** | **Condition** | **Administration** | **Phase** | **Clinical trial identifier** |
| --- | --- | --- | --- | --- |
| Daptomycin | Bacterial skin infections | Intravenous | Approved | NCT01211470 |
| Human lactoferrin peptide 1-11 | Bacterial infections and mycoses | Intravenous | Approved | NCT00509938 |
| Vancomycin | *Staphylococcal* infections | Intravenous | Approved | NCT00175370 |
| PAC113 | Oral candidiasis | Mouthrinse | Phase II | NCT00659971 |
| Bacitracin | Skin infection | External | Phase IV | NCT03929224 |
| Colistin | Multidrug-resistance Gram-negative infections | Intravenous | Phase IV | NCT03397914 |
| LL-37 | Melanoma | Intratumoral | Phase I | NCT02225366 |
| LTX-315 | Solid tumors | Intratumoral | Phase I | NCT01986426 |
| ANG1005 | Breast cancer  brain metastases | Intravenous | Phase II | NCT02048059 |

*PAC113* an active segment of histatin 5; *LL-37* leucine-leucine-37; *LTX-315* a chemically modified 9-mer cationic peptide; *ANG1005* a brain penetrating peptide-drug conjugate

**References**

1. Rea MC, Sit CS, Clayton E, O'Connor PM, Whittal RM, Zheng J*, et al*. Thuricin CD, a posttranslationally modified bacteriocin with a narrow spectrum of activity against Clostridium difficile. Proc Natl Acad Sci U S A. 2010;107(20):9352-7.

2. Rosengren KJ, Clark RJ, Daly NL, Goransson U, Jones A, Craik DJ. Microcin J25 has a threaded sidechain-to-backbone ring structure and not a head-to-tail cyclized backbone. J Am Chem Soc. 2003;125(41):12464-74.

3. Helynck G, Dubertret C, Mayaux JF, Leboul J. Isolation of RP 71955, a new anti-HIV-1 peptide secondary metabolite. J Antibiot (Tokyo). 1993;46(11):1756-7.

4. Plan MR, Goransson U, Clark RJ, Daly NL, Colgrave ML, Craik DJ. The cyclotide fingerprint in oldenlandia affinis: elucidation of chemically modified, linear and novel macrocyclic peptides. Chembiochem. 2007;8(9):1001-11.

5. Games PD, Dos Santos IS, Mello EO, Diz MS, Carvalho AO, de Souza-Filho GA*, et al*. Isolation, characterization and cloning of a cDNA encoding a new antifungal defensin from *Phaseolus vulgaris* *L.* seeds. Peptides. 2008;29(12):2090-100.

6. Barry DG, Daly NL, Bokesch HR, Gustafson KR, Craik DJ. Solution structure of the cyclotide palicourein: implications for the development of a pharmaceutical framework. Structure. 2004;12(1):85-94.

7. Lai R, Lomas LO, Jonczy J, Turner PC, Rees HH. Two novel non-cationic defensin-like antimicrobial peptides from haemolymph of the female tick, Amblyomma hebraeum. Biochem J. 2004;379(Pt 3):681-5.

8. Gauri SS, Mandal SM, Pati BR, Dey S. Purification and structural characterization of a novel antibacterial peptide from Bellamya bengalensis: activity against ampicillin and chloramphenicol resistant Staphylococcus epidermidis. Peptides. 2011;32(4):691-6.

9. Lai R, Liu H, Hui Lee W, Zhang Y. An anionic antimicrobial peptide from toad Bombina maxima. Biochem Biophys Res Commun. 2002;295(4):796-9.

10. Strub JM, Goumon Y, Lugardon K, Capon C, Lopez M, Moniatte M*, et al*. Antibacterial activity of glycosylated and phosphorylated chromogranin A-derived peptide 173-194 from bovine adrenal medullary chromaffin granules. J Biol Chem. 1996;271(45):28533-40.

11. Comb M, Herbert E, Crea R. Partial characterization of the mRNA that codes for enkephalins in bovine adrenal medulla and human pheochromocytoma. Proc Natl Acad Sci U S A. 1982;79(2):360-4.

12. Jung HH, Yang ST, Sim JY, Lee S, Lee JY, Kim HH*, et al*. Analysis of the solution structure of the human antibiotic peptide dermcidin and its interaction with phospholipid vesicles. BMB Rep. 2010;43(5):362-8.

13. Soscia SJ, Kirby JE, Washicosky KJ, Tucker SM, Ingelsson M, Hyman B*, et al*. The Alzheimer's disease-associated amyloid beta-protein is an antimicrobial peptide. PLoS One. 2010;5(3):e9505.

14. Kim JY, Park SC, Lee JK, Choi SJ, Hahm KS, Park Y. Novel antibacterial activity of beta(2)-microglobulin in human amniotic fluid. PLoS One. 2012;7(11):e47642.

15. Hultmark D, Engstrom A, Bennich H, Kapur R, Boman HG. Insect immunity: isolation and structure of cecropin D and four minor antibacterial components from Cecropia pupae. Eur J Biochem. 1982;127(1):207-17.

16. Marchini D, Giordano PC, Amons R, Bernini LF, Dallai R. Purification and primary structure of ceratotoxin A and B, two antibacterial peptides from the female reproductive accessory glands of the medfly Ceratitis capitata (Insecta:Diptera). Insect Biochem Mol Biol. 1993;23(5):591-8.

17. Argiolas A, Pisano JJ. Isolation and characterization of two new peptides, mastoparan C and crabrolin, from the venom of the European hornet, Vespa crabro. J Biol Chem. 1984;259(16):10106-11.

18. Vlasak R, Unger-Ullmann C, Kreil G, Frischauf AM. Nucleotide sequence of cloned cDNA coding for honeybee prepromelittin. Eur J Biochem. 1983;135(1):123-6.

19. Cole AM, Weis P, Diamond G. Isolation and characterization of pleurocidin, an antimicrobial peptide in the skin secretions of winter flounder. J Biol Chem. 1997;272(18):12008-13.

20. Iijima N, Tanimoto N, Emoto Y, Morita Y, Uematsu K, Murakami T*, et al*. Purification and characterization of three isoforms of chrysophsin, a novel antimicrobial peptide in the gills of the red sea bream, Chrysophrys major. Eur J Biochem. 2003;270(4):675-86.

21. Yi GS, Park CB, Kim SC, Cheong C. Solution structure of an antimicrobial peptide buforin II. FEBS Lett. 1996;398(1):87-90.

22. Conlon JM, Kolodziejek J, Nowotny N, Leprince J, Vaudry H, Coquet L*, et al*. Characterization of antimicrobial peptides from the skin secretions of the Malaysian frogs, Odorrana hosii and Hylarana picturata (Anura:Ranidae). Toxicon. 2008;52(3):465-73.

23. Zasloff M. Magainins, a class of antimicrobial peptides from Xenopus skin: isolation, characterization of two active forms, and partial cDNA sequence of a precursor. Proc Natl Acad Sci U S A. 1987;84(15):5449-53.

24. Santana CJC, Magalhaes ACM, Prias-Marquez CA, Falico DA, Dos Santos Junior ACM, Lima BD*, et al*. Biological properties of a novel multifunctional host defense peptide from the skin secretion of the Chaco Tree Frog, *Boana raniceps*. Biomolecules. 2020;10(5):790.

25. Gallo RL, Kim KJ, Bernfield M, Kozak CA, Zanetti M, Merluzzi L*, et al*. Identification of CRAMP, a cathelin-related antimicrobial peptide expressed in the embryonic and adult mouse. J Biol Chem. 1997;272(20):13088-93.

26. Cho HS, Yum J, Lariviere A, Leveque N, Le QVC, Ahn B*, et al*. Opossum cathelicidins exhibit antimicrobial activity against a broad spectrum of pathogens including West Nile virus. Front Immunol. 2020;11:347.

27. Chen C, Brock R, Luh F, Chou PJ, Larrick JW, Huang RF*, et al*. The solution structure of the active domain of CAP18-a lipopolysaccharide binding protein from rabbit leukocytes. FEBS Lett. 1995;370(1-2):46-52.

28. Mahoney MM, Lee AY, Brezinski-Caliguri DJ, Huttner KM. Molecular analysis of the sheep cathelin family reveals a novel antimicrobial peptide. FEBS Lett. 1995;377(3):519-22.

29. Yang S, Lee CW, Kim HJ, Jung HH, Kim JI, Shin SY*, et al*. Structural analysis and mode of action of BMAP-27, a cathelicidin-derived antimicrobial peptide. Peptides. 2019;118:170106.

30. Sipos D, Andersson M, Ehrenberg A. The structure of the mammalian antibacterial peptide cecropin P1 in solution, determined by proton-NMR. Eur J Biochem. 1992;209(1):163-9.

31. Cole AM, Kim YH, Tahk S, Hong T, Weis P, Waring AJ*, et al*. Calcitermin, a novel antimicrobial peptide isolated from human airway secretions. FEBS Lett. 2001;504(1-2):5-10.

32. Zelezetsky I, Pontillo A, Puzzi L, Antcheva N, Segat L, Pacor S*, et al*. Evolution of the primate cathelicidin. Correlation between structural variations and antimicrobial activity. J Biol Chem. 2006;281(29):19861-71.

33. Abraham T, Prenner EJ, Lewis RN, Mant CT, Keller S, Hodges RS*, et al*. Structure-activity relationships of the antimicrobial peptide gramicidin S and its analogs: aqueous solubility, self-association, conformation, antimicrobial activity and interaction with model lipid membranes. Biochim Biophys Acta. 2014;1838(5):1420-9.

34. Martinez B, Suarez JE, Rodriguez A. Lactococcin 972 : a homodimeric lactococcal bacteriocin whose primary target is not the plasma membrane. Microbiology (Reading). 1996;142 ( Pt 9):2393-8.

35. Mygind PH, Fischer RL, Schnorr KM, Hansen MT, Sonksen CP, Ludvigsen S*, et al*. Plectasin is a peptide antibiotic with therapeutic potential from a saprophytic fungus. Nature. 2005;437(7061):975-80.

36. Weber AN, Tauszig-Delamasure S, Hoffmann JA, Lelievre E, Gascan H, Ray KP*, et al*. Binding of the Drosophila cytokine Spatzle to Toll is direct and establishes signaling. Nat Immunol. 2003;4(8):794-800.

37. Laederach A, Andreotti AH, Fulton DB. Solution and micelle-bound structures of tachyplesin I and its active aromatic linear derivatives. Biochemistry. 2002;41(41):12359-68.

38. Cuesta A, Meseguer J, Esteban MA. The antimicrobial peptide hepcidin exerts an important role in the innate immunity against bacteria in the bony fish gilthead seabream. Mol Immunol. 2008;45(8):2333-42.

39. Lynn DJ, Higgs R, Gaines S, Tierney J, James T, Lloyd AT*, et al*. Bioinformatic discovery and initial characterisation of nine novel antimicrobial peptide genes in the chicken. Immunogenetics. 2004;56(3):170-7.

40. Evans EW, Beach GG, Wunderlich J, Harmon BG. Isolation of antimicrobial peptides from avian heterophils. J Leukoc Biol. 1994;56(5):661-5.

41. Bauer F, Schweimer K, Kluver E, Conejo-Garcia JR, Forssmann WG, Rosch P*, et al*. Structure determination of human and murine beta-defensins reveals structural conservation in the absence of significant sequence similarity. Protein Sci. 2001;10(12):2470-9.

42. Aumelas A, Mangoni M, Roumestand C, Chiche L, Despaux E, Grassy G*, et al*. Synthesis and solution structure of the antimicrobial peptide protegrin-1. Eur J Biochem. 1996;237(3):575-83.

43. Tran D, Tran PA, Tang YQ, Yuan J, Cole T, Selsted ME. Homodimeric theta-defensins from rhesus macaque leukocytes: isolation, synthesis, antimicrobial activities, and bacterial binding properties of the cyclic peptides. J Biol Chem. 2002;277(5):3079-84.

44. Liu L, Wang L, Jia HP, Zhao C, Heng HH, Schutte BC*, et al*. Structure and mapping of the human beta-defensin HBD-2 gene and its expression at sites of inflammation. Gene. 1998;222(2):237-44.

45. Del Pero M, Boniotto M, Zuccon D, Cervella P, Spano A, Amoroso A*, et al*. Beta-defensin 1 gene variability among non-human primates. Immunogenetics. 2002;53(10-11):907-13.

46. Ghosh D, Porter E, Shen B, Lee SK, Wilk D, Drazba J*, et al*. Paneth cell trypsin is the processing enzyme for human defensin-5. Nat Immunol. 2002;3(6):583-90.

47. Torrent M, Badia M, Moussaoui M, Sanchez D, Nogues MV, Boix E. Comparison of human RNase 3 and RNase 7 bactericidal action at the Gram-negative and Gram-positive bacterial cell wall. FEBS J. 2010;277(7):1713-25.

48. Simon A, Kullberg BJ, Tripet B, Boerman OC, Zeeuwen P, van der Ven-Jongekrijg J*, et al*. Drosomycin-like defensin, a human homologue of Drosophila melanogaster drosomycin with antifungal activity. Antimicrob Agents Chemother. 2008;52(4):1407-12.

49. Cheung-Lee WL, Parry ME, Jaramillo Cartagena A, Darst SA, Link AJ. Discovery and structure of the antimicrobial lasso peptide citrocin. J Biol Chem. 2019;294(17):6822-30.

50. Qutb AM, Wei F, Dong W. Prediction and characterization of cationic arginine-rich plant antimicrobial peptide SM-985 from teosinte (Zea mays ssp. mexicana). Front Microbiol. 2020;11:1353.

51. Chernysh S, Kim SI, Bekker G, Pleskach VA, Filatova NA, Anikin VB*, et al*. Antiviral and antitumor peptides from insects. Proc Natl Acad Sci U S A. 2002;99(20):12628-32.

52. Zahn M, Berthold N, Kieslich B, Knappe D, Hoffmann R, Strater N. Structural studies on the forward and reverse binding modes of peptides to the chaperone DnaK. J Mol Biol. 2013;425(14):2463-79.

53. Xie Y, Wan H, Zeng X, Zhang Z, Wang Y. Characterization and antimicrobial evaluation of a new Spgly-AMP, glycine-rich antimicrobial peptide from the mud crab Scylla paramamosain. Fish Shellfish Immunol. 2020;106:384-92.

54. de Jesus Oliveira T, Oliveira UC, da Silva Junior PI. Serrulin: A glycine-rich bioactive peptide from the hemolymph of the yellow tityus serrulatus scorpion. Toxins (Basel). 2019;11(9):517.

55. Roncevic T, Cikes-Culic V, Maravic A, Capanni F, Gerdol M, Pacor S*, et al*. Identification and functional characterization of the astacidin family of proline-rich host defence peptides (PcAst) from the red swamp crayfish (Procambarus clarkii, Girard 1852). Dev Comp Immunol. 2020;105:103574.

56. Silphaduang U, Noga EJ. Peptide antibiotics in mast cells of fish. Nature. 2001;414(6861):268-9.

57. Wang L, Chan JY, Rego JV, Chong CM, Ai N, Falcao CB*, et al*. Rhodamine B-conjugated encrypted vipericidin nonapeptide is a potent toxin to zebrafish and associated with in vitro cytotoxicity. Biochim Biophys Acta. 2015;1850(6):1253-60.

58. Mardirossian M, Perebaskine N, Benincasa M, Gambato S, Hofmann S, Huter P*, et al*. The dolphin proline-rich antimicrobial peptide Tur1A inhibits protein synthesis by targeting the bacterial ribosome. Cell Chem Biol. 2018;25(5):530-9 e7.

59. Cho HS, Soundrarajan N, Le Van Chanh Q, Jeon H, Cha SY, Kang M*, et al*. The novel cathelicidin of naked mole rats, Hg-CATH, showed potent antimicrobial activity and low cytotoxicity. Gene. 2018;676:164-70.

60. Gudmundsson GH, Magnusson KP, Chowdhary BP, Johansson M, Andersson L, Boman HG. Structure of the gene for porcine peptide antibiotic PR-39, a cathelin gene family member: comparative mapping of the locus for the human peptide antibiotic FALL-39. Proc Natl Acad Sci U S A. 1995;92(15):7085-9.

61. Harwig SS, Kokryakov VN, Swiderek KM, Aleshina GM, Zhao C, Lehrer RI. Prophenin-1, an exceptionally proline-rich antimicrobial peptide from porcine leukocytes. FEBS Lett. 1995;362(1):65-9.

62. Rozek A, Friedrich CL, Hancock RE. Structure of the bovine antimicrobial peptide indolicidin bound to dodecylphosphocholine and sodium dodecyl sulfate micelles. Biochemistry. 2000;39(51):15765-74.

63. Oppenheim FG, Xu T, McMillian FM, Levitz SM, Diamond RD, Offner GD*, et al*. Histatins, a novel family of histidine-rich proteins in human parotid secretion. Isolation, characterization, primary structure, and fungistatic effects on Candida albicans. J Biol Chem. 1988;263(16):7472-7.

64. Toda H, Williams JA, Gulledge M, Sehgal A. A sleep-inducing gene, nemuri, links sleep and immune function in Drosophila. Science. 2019;363(6426):509-15.

65. Qin CL, Huang W, Zhou SQ, Wang XC, Liu HH, Fan MH*, et al*. Characterization of a novel antimicrobial peptide with chitin-biding domain from Mytilus coruscus. Fish Shellfish Immunol. 2014;41(2):362-70.

66. Fernandes JM, Kemp GD, Molle MG, Smith VJ. Anti-microbial properties of histone H2A from skin secretions of rainbow trout, Oncorhynchus mykiss. Biochem J. 2002;368(Pt 2):611-20.

67. Shi Y, Li C, Wang M, Chen Z, Luo Y, Xia XS*, et al*. Cathelicidin-DM is an antimicrobial peptide from Duttaphrynus melanostictus and Has wound-healing therapeutic potential. ACS Omega. 2020;5(16):9301-10.

68. Feng X, Jin S, Wang M, Pang Q, Liu C, Liu R*, et al*. The critical role of tryptophan in the antimicrobial activity and cell toxicity of the duck antimicrobial peptide DCATH. Front Microbiol. 2020;11:1146.

69. Froidevaux R, Krier F, Nedjar-Arroume N, Vercaigne-Marko D, Kosciarz E, Ruckebusch C*, et al*. Antibacterial activity of a pepsin-derived bovine hemoglobin fragment. FEBS Lett. 2001;491(1-2):159-63.

70. Chapple DS, Hussain R, Joannou CL, Hancock RE, Odell E, Evans RW*, et al*. Structure and association of human lactoferrin peptides with Escherichia coli lipopolysaccharide. Antimicrob Agents Chemother. 2004;48(6):2190-8.

71. Davie EW, Kulman JD. An overview of the structure and function of thrombin. Semin Thromb Hemost. 2006;32 Suppl 1:3-15.

72. Hou J, Liu Z, Cao S, Wang H, Jiang C, Hussain MA*, et al*. Broad-spectrum antimicrobial activity and low cytotoxicity against human cells of a peptide derived from bovine alphaS1-casein. Molecules. 2018;23(5):1220.

73. Briolat J, Wu SD, Mahata SK, Gonthier B, Bagnard D, Chasserot-Golaz S*, et al*. New antimicrobial activity for the catecholamine release-inhibitory peptide from chromogranin A. Cell Mol Life Sci. 2005;62(3):377-85.

74. Bjerkan L, Schreurs O, Engen SA, Jahnsen FL, Baekkevold ES, Blix IJ*, et al*. The short form of TSLP is constitutively translated in human keratinocytes and has characteristics of an antimicrobial peptide. Mucosal Immunol. 2015;8(1):49-56.

75. Huang TC, Lee JF, Chen JY. Pardaxin, an antimicrobial peptide, triggers caspase-dependent and ROS-mediated apoptosis in HT-1080 cells. Mar Drugs. 2011;9(10):1995-2009.

76. Han YY, Liu HY, Han DJ, Zong XC, Zhang SQ, Chen YQ. Role of glycosylation in the anticancer activity of antibacterial peptides against breast cancer cells. Biochem Pharmacol. 2013;86(9):1254-62.

77. Li RF, Lu YL, Lu YB, Zhang HR, Huang L, Yin Y*, et al*. Antiproliferative effect and characterization of a novel antifungal peptide derived from human Chromogranin A. Exp Ther Med. 2015;10(6):2289-94.

78. Falcao CB, Perez-Peinado C, de la Torre BG, Mayol X, Zamora-Carreras H, Jimenez MA*, et al*. Structural dissection of crotalicidin, a rattlesnake venom cathelicidin, retrieves a fragment with antimicrobial and antitumor activity. J Med Chem. 2015;58(21):8553-63.

79. Chen X, Zhang L, Ma C, Zhang Y, Xi X, Wang L*, et al*. A novel antimicrobial peptide, Ranatuerin-2PLx, showing therapeutic potential in inhibiting proliferation of cancer cells. Biosci Rep. 2018;38(6):BSR20180710.

80. Ting CH, Liu YC, Lyu PC, Chen JY. Nile tilapia derived antimicrobial peptide TP4 exerts antineoplastic activity through microtubule disruption. Mar Drugs. 2018;16(12):462.

81. Lehmann J, Retz M, Sidhu SS, Suttmann H, Sell M, Paulsen F*, et al*. Antitumor activity of the antimicrobial peptide magainin II against bladder cancer cell lines. Eur Urol. 2006;50(1):141-7.

82. Li M, Xi X, Ma C, Chen X, Zhou M, Burrows JF*, et al*. A novel dermaseptin isolated from the skin secretion of phyllomedusa tarsius and its cationicity-enhanced analogue exhibiting effective antimicrobial and anti-proliferative activities. Biomolecules. 2019;9(10):628.

83. Baindara P, Gautam A, Raghava GPS, Korpole S. Anticancer properties of a defensin like class IId bacteriocin Laterosporulin10. Sci Rep. 2017;7:46541.

84. Flores-Alvarez LJ, Guzman-Rodriguez JJ, Lopez-Gomez R, Salgado-Garciglia R, Ochoa-Zarzosa A, Lopez-Meza JE. PaDef defensin from avocado (Persea americana var. drymifolia) is cytotoxic to K562 chronic myeloid leukemia cells through extrinsic apoptosis. Int J Biochem Cell Biol. 2018;99:10-8.

85. Gong X, Zou L, Wang M, Zhang Y, Peng S, Zhong M*, et al*. Gramicidin inhibits cholangiocarcinoma cell growth by suppressing EGR4. Artif Cells Nanomed Biotechnol. 2020;48(1):53-9.

86. Hansen IKO, Isaksson J, Poth AG, Hansen KO, Andersen AJC, Richard CSM*, et al*. Isolation and characterization of antimicrobial peptides with unusual disulfide connectivity from the colonial ascidian synoicum turgens. Mar Drugs. 2020;18(1):51.

87. Swithenbank L, Cox P, Harris LG, Dudley E, Sinclair K, Lewis P*, et al*. Temporin A and Bombinin H2 antimicrobial peptides exhibit selective cytotoxicity to lung cancer cells. Scientifica (Cairo). 2020;2020:3526286.

88. Tang C, Shao X, Sun B, Huang W, Qiu F, Chen Y*, et al*. Anticancer mechanism of peptide P18 in human leukemia K562 cells. Org Biomol Chem. 2010;8(5):984-7.

89. Ikonomopoulou MP, Fernandez-Rojo MA, Pineda SS, Cabezas-Sainz P, Winnen B, Morales RAV*, et al*. Gomesin inhibits melanoma growth by manipulating key signaling cascades that control cell death and proliferation. Sci Rep. 2018;8(1):11519.

90. Ohtake T, Fujimoto Y, Ikuta K, Saito H, Ohhira M, Ono M*, et al*. Proline-rich antimicrobial peptide, PR-39 gene transduction altered invasive activity and actin structure in human hepatocellular carcinoma cells. Br J Cancer. 1999;81(3):393-403.

91. Norouzi Z, Salimi A, Halabian R, Fahimi H. Nisin, a potent bacteriocin and anti-bacterial peptide, attenuates expression of metastatic genes in colorectal cancer cell lines. Microb Pathog. 2018;123:183-9.

92. Xia L, Wu Y, Kang S, Ma J, Yang J, Zhang F. CecropinXJ, a silkworm antimicrobial peptide, induces cytoskeleton disruption in esophageal carcinoma cells. Acta Biochim Biophys Sin (Shanghai). 2014;46(10):867-76.

93. Figueira TN, Oliveira FD, Almeida I, Mello EO, Gomes VM, Castanho M*, et al*. Challenging metastatic breast cancer with the natural defensin PvD1. Nanoscale. 2017;9(43):16887-99.

94. Xu P, Lv D, Wang X, Wang Y, Hou C, Gao K*, et al*. Inhibitory effects of Bombyx mori antimicrobial peptide cecropins on esophageal cancer cells. Eur J Pharmacol. 2020;887:173434.

95. Chen YF, Shih PC, Kuo HM, Yang SN, Lin YY, Chen WF*, et al*. TP3, an antimicrobial peptide, inhibits infiltration and motility of glioblastoma cells via modulating the tumor microenvironment. Cancer Med. 2020;9(11):3918-31.

96. Berge G, Eliassen LT, Camilio KA, Bartnes K, Sveinbjornsson B, Rekdal O. Therapeutic vaccination against a murine lymphoma by intratumoral injection of a cationic anticancer peptide. Cancer Immunol Immunother. 2010;59(8):1285-94.

97. Li D, Wang W, Shi HS, Fu YJ, Chen X, Chen XC*, et al*. Gene therapy with beta-defensin 2 induces antitumor immunity and enhances local antitumor effects. Hum Gene Ther. 2014;25(1):63-72.
